# Efficient Composite Infrared Spectroscopy: Combining the Double-Harmonic Approximation with Machine Learning Potentials

**DOI:** 10.1021/acs.jctc.4c01157

**Published:** 2024-12-12

**Authors:** Philipp Pracht, Yuthika Pillai, Venkat Kapil, Gábor Csányi, Nils Gönnheimer, Martin Vondrák, Johannes T. Margraf, David J. Wales

**Affiliations:** † Yusuf Hamied Department of Chemistry, University of Cambridge, Lensfield Road, Cambridge CB2 1EW, U.K.; ‡ Department of Chemical Engineering, Massachusetts Institute of Technology, Cambridge, Massachusetts 02139, United States; § Department of Physics and Astronomy, University College London, 17-19 Gordon Street, London WC1H 0AH, U.K.; ∥ Thomas Young Centre & London Centre for Nanotechnology, 19 Gordon Street, London WC1H 0AH, U.K.; ⊥ Engineering Laboratory, University of Cambridge, Trumpington Street, Cambridge CB2 1PZ, U.K.; # University of Bayreuth, Bavarian Center for Battery Technology (BayBatt), 95448 Bayreuth, Germany

## Abstract

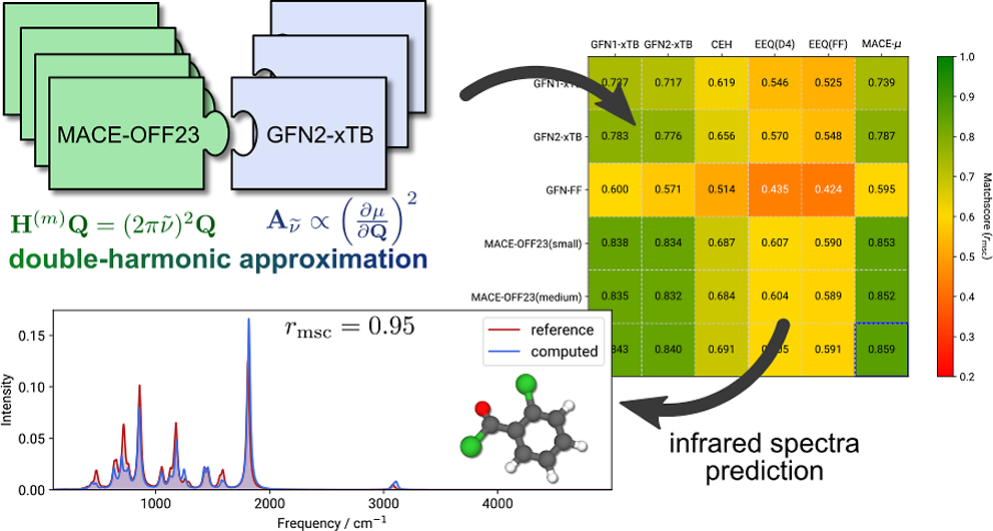

Vibrational spectroscopy is a cornerstone technique for molecular characterization and offers an ideal target for the computational investigation of molecular materials. Building on previous comprehensive assessments of efficient methods for infrared (IR) spectroscopy, this study investigates the predictive accuracy and computational efficiency of gas-phase IR spectra calculations, accessible through a combination of modern semiempirical quantum mechanical and transferable machine learning potentials. A composite approach for IR spectra prediction based on the double-harmonic approximation, utilizing harmonic vibrational frequencies in combination squared derivatives of the molecular dipole moment, is employed. This approach allows for methodical flexibility in the calculation of IR intensities from molecular dipoles and the corresponding vibrational modes. Various methods are systematically tested to suggest a suitable protocol with an emphasis on computational efficiency. Among these methods, semiempirical extended tight-binding (xTB) models, classical charge equilibrium models, and machine learning potentials trained for dipole moment prediction are assessed across a diverse data set of organic molecules. We particularly focus on the recently reported foundational machine learning potential MACE-OFF23 to address the accuracy limitations of conventional low-cost quantum mechanical and force-field methods. This study aims to establish a standard for the efficient computational prediction of IR spectra, facilitating the rapid and reliable identification of unknown compounds and advancing automated high-throughput analytical workflows in chemistry.

## 1 Introduction

Infrared (IR) spectroscopy remains a key analytical tool for characterizing molecular structures and dynamics, widely applied from fundamental research to industrial quality control.^1−3^ Among the various types of vibrational spectroscopy, IR spectroscopy is arguably most widely adapted and has hence been a longstanding target for computational simulations, where the two most common approaches are Fourier transform-based spectra prediction from both classical and quantum dynamical simulations,^4^ or static approaches based on the harmonic molecular Hessian and the so-called double-harmonic approximation (DHA).^5^ While the former approach includes anharmonic effects and an averaging over conformational states via time evolution of the system, the static approach generally produces less computational overhead. This computational efficiency can be especially important, since predicting vibrational spectra often involves time-consuming first-principles calculations that account specifically for electronic structure. To partially circumvent these problems, several approaches have been proposed to predict vibrational spectra from dynamical simulations via machine learning.^6−11^ Within static approaches, the current state-of-the-art corresponds to extensive methods like VPT2,^12,13^ that go beyond the harmonic approximation, include electronic effects, and correctly treat vibrational anharmonicity. While these approaches are routinely applicable with via a variety of theories implemented in software program packages, they are often associated with increased computational effort, in particular for larger system size,^14^ and thus are less suitable for high-throughput applications than more approximate methods. The static DHA approaches have the advantage that (only) 6*N*_atoms_ potential evaluations are required to set up a seminumerical Hessian matrix, rather than several ps of molecular dynamics.^4,5^ Additionally, analytical formulations for second derivatives may be available, further reducing the computation time compared to such seminumerical approaches.^15^ Unsurprisingly, the demand for quicker and more cost-effective computational approaches has led to significant developments in semiempirical quantum mechanical (SQM)^16,17^ and molecular machine learning potentials (MLPs).^18−23^ These two contemporary families of methodology share similar aims and designs, serving as computationally efficient alternatives to the aforementioned first-principles calculations. Importantly, such methods are distinct from the direct deep neural network-based prediction of IR spectra,^24^ and provide a more conventional integration into computational workflows. In this framework, advances in MLPs or SQM methods can easily be adapted, and promise greater flexibility in treating a diverse range of molecular and condensed phase systems.

**Figure 1:**
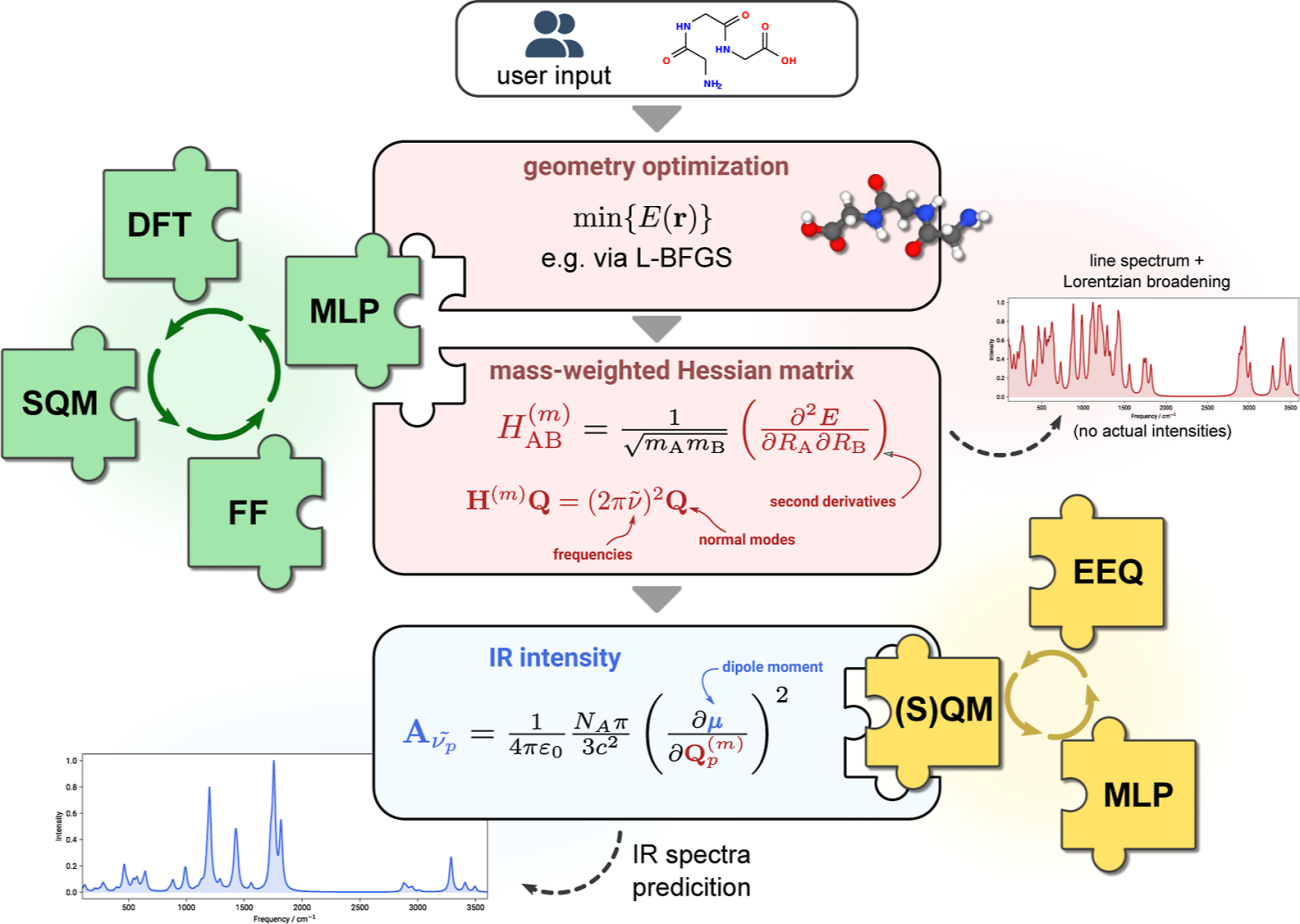
Composite combination of methods for IR spectra calculation within the double-harmonic approximation.

Building upon our previous evaluations of semiempirical extended tight-binding and machine learning approaches for vibrational spectra,^9,11,25^ this study introduces a “plug-in” composite strategy (cf. Figure 1) designed to enhance the speed and accuracy of IR spectral predictions and provide a snapshot of the current DHA approach’s capabilities. By combining harmonically approximated frequencies, i.e., from diagonalization of the mass-weighted Hessian, with molecular dipole moment derivatives calculated via diverse computational techniques, the goal is to achieve a balanced approach that optimizes both computational efficiency and predictive fidelity. Central to our methodological framework is the integration of a recently reported MLP, MACE-OFF23^26^ for the computation of molecular frequencies. This model employs the MACE^27^ MLP architecture for organic molecules based on accurate numerical data, promising to bridge the accuracy gap typically associated with faster computational methods. As a foundational MLP, a key expectation for MACE-OFF23 is usability without further retraining, similar to the use of a conventional QM or SQM method. Unfortunately, the current implementation of this MLP does not include molecular electrostatics and so obtaining a dipole moment is not possible using only MACE-OFF23. Therefore, a separate MACE model is introduced and trained for dipole moment prediction. The kQEq model^28,29^ was also considered. We compare MLP-derived dipole moments with those obtained from classical atomic charge models and more traditional quantum mechanically derived dipole moments, providing a comprehensive evaluation of the performance for each method. Additionally, the ability to obtain the correct molecular geometries, i.e., find the minima on the potential energy landscape via optimization procedures,^30,31^ plays a crucial role in a composite approach, influencing both frequency calculations and dipole moment predictions. By assessing method performance across these method capabilities, we not only seek to validate the utility of MLPs in theoretical spectroscopy, but also explore their integration into established computational workflows. Some further key targets are to investigate whether ML methods trained on the SPICE data set^32^ are suitable surrogates for predictions at DFT-level accuracy, and whether their composite combination with other, for example SQM methods can advance our predictive capabilities in general. As such, we do not target retraining of the existing MACE-OFF23 model and rather investigate its out-of-the-box performance, similar to a SQM or QM method.

With these targets in mind, we aim to advance the practical application of vibrational spectroscopy, enabling rapid and reliable identification of compounds with significantly reduced computational demands compared to conventional electronic structure calculations. A short summary is first provided of IR spectra computation in the static harmonic approach and the DHA. The performance of the component techniques required for calculating vibrational spectra, namely geometry optimization, dipole moment, and frequency prediction, are evaluated based on a selection of benchmark sets from the literature. Finally, IR spectra are computed putting these properties together, combining different levels of theory in a composite approach.

## 2 Theory

### 2.1 Double-Harmonic Approximation

In the harmonic approach, molecular vibrations are derived from second derivatives of the energy *E* with respect to the nuclear positions *R*_A/B_
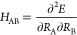
1where **H** is the Hessian matrix. The mass-weighted force constant matrix (FCM) **F**^(*m*)^ is obtained from
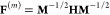
2where **M** is the diagonal matrix of the atomic masses. Diagonalization of **F**^(*m*)^ according to
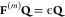
3provides a set of 3*N*_at_ eigenvectors *Q*_p_^(*m*)^, which correspond to the normal modes in Cartesian space, and their corresponding eigenvalues ϵ. To obtain frequencies in reciprocal centimeters  for mode p, eigenvalue ϵ_p_ is converted with
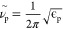
4Note that alternative formulations with molecular representations in internal curvilinear coordinates are available. These formulations can be more efficient than alternative formulations for Hessian calculations and geometry optimizations,^33−36^ but for simplicity we refer to the plain Cartesian formulation. In the so-called “double-harmonic approximation”, which gets its name from the Taylor series truncation after the quadratic terms for both frequencies and dipole derivatives,^5,37^ the intensity  for the normal mode *p* is assumed proportional to the squared dipole derivative of the corresponding normal mode and the integral absorption coefficient is then given as
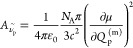
5where  corresponds to the derivative of the dipole moment with respect to the normal mode coordinates, *N*_*A*_ is Avogadro’s constant, *c* is the speed of light, and ε_0_ is the vacuum permittivity. In practice, the derivative of the dipole moment is evaluated with respect to the Cartesian coordinates of each atom *j* and is afterward transformed onto the coordinates of the vibrational normal mode *p* according to
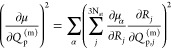
6in which α ∈ (*x*,*y*,*z*) are the components of the dipole moment gradient in Cartesian coordinates. The combination of the vibrational frequency  of mode p and the corresponding intensity in terms of the integral absorption coefficient  determines the calculated vibrational spectrum. The double-harmonic approximation herein leads to errors of the spectrum in both frequencies and intensities due to truncation of higher order terms of the respective Taylor series.^5^ However, the accuracy of those two quantities can be improved independently, as discussed below. Although the combination of both quantities via the DHA is a standard procedure in modern quantum chemistry codes, to the best of our knowledge only a few codes allow the explicit definition of separate methods for frequency and dipole calculation and even fewer methods exist that are explicitly designed for a composite use. For a recent example, we refer the reader to the recently introduced PTB method.^38^

### 2.2 Computational Details

For the computation of molecular dipole moments, molecular geometries, frequencies and IR spectra, we generally refer to a benchmark set of 7193 gas-phase molecules^25^ ranging from three to 77 atoms in size, and containing the elements H, C, N, O, F, Cl, Br, S, and P, which coincides with the elements available in MACE-OFF23.^26^ For simplicity, we refer to this set as “IR7193” in the following. The corresponding molecules can generally be regarded as “drug-like” with regards to the average size and chemical composition. This set was originally composed^25^ simply by obtaining all freely available experimental gas-phase reference IR spectra and the corresponding molecular structures from the NIST database^39^ which were then compared with computed spectra for the semiempirical GFN*n*-xTB approach,^17^ the force-field GFN-FF,^40^ and DFT B3LYP-3c (an abbreviation for B3LYP-D3(BJ)^ATM^-gCP/def2-mSVP^41−45^). B3LYP-3c herein was identified as a well-balanced DFT level of theory for IR spectra computation and can serve as a theoretical reference for testing other low-cost methods. To avoid additional conformational effects,^25^ and effects of possible experimental measurement impurities or conditions like temperature and pressure, we take the readily available theoretical gas-phase spectra calculated at B3LYP-3c level as our reference. For completeness, comparisons with experimental gas phase IR data from the NIST database are summarized in Section S7 of the Supporting Information. All calculated reference B3LYP-3c spectra were taken from ref (25). New DFT calculations at the B3LYP-3c and ωB97M-D3(BJ) levels of theory were only performed to obtain molecular geometries and molecular dipole moments, and used ORCA 5.0.4.^46,47^

Seminumerical Hessian calculations at the GFN1-xTB,^48^ GFN2-xTB,^49^ and GFN-FF^40^ levels of theory were performed with the CREST program.^50,51^ The same program was used to simultaneously obtain derivatives of the molecular dipole moment with an empirical charge equilibrium (EEQ) model using parameters employed in the D4 dispersion correction, as well as dipole derivatives obtained from the so-called Charge Extended Hückel (CEH) model, published for the charge-adaptive q-vSZP basis set.^52^ Installation instructions for the MACE suite can be found at https://github.com/ACEsuit/mace. MACE-OFF23 calculations were performed using the ASE interface.^53^ The associated geometry optimizations were done with ASE’s implementation of the L-BFGS algorithm and a root-mean-square (RMS) force convergence criterion of 1.0 × 10^–4^*E*_h_/*a*_0_. Seminumerical calculations of the corresponding Hessian and dipole derivatives were performed with a standalone script employing coordinate displacements of 1.0 × 10^–3^*a*_0_. The seminumerical Hessian calculations of MACE-OFF23 were also compared with a new analytical (“autograd”) implementation. No reparametrization was attempted for MACE-OFF23 based on the present data set because our study targets the use of the MLP for a pretrained foundational model, similar to a conventional SQM or QM method.

Analysis tools for the combination of computed Hessian matrices and dipole derivatives into IR spectra can be found at https://github.com/pprcht/vibtools. Our comparison of IR spectra is based on a similarity “match”score *r*_msc_ based on the Cauchy-Schwarz inequality as defined by eq S3 in the Supporting Information and can have values from 0 (no match) to 1 (perfect correlation). Any such metric is heavily dependent on the line shape employed to broaden the calculated “stick” spectrum. Here, by default we employ Lorentzian functions with a full width at half-maximum (fwhm) of 30 cm^–1^, which in correspondence with previous studies^25^ is chosen slightly larger than the estimated NIST average bandwidth^54^ of 24 cm^–1^ to allow more leeway in accounting for noise in the experimental spectra. For more information on spectra comparison with this metric and alternative measures (Euclidean norm *r*_euc_, the Spearman correlation coefficient *r*_scc_, Pearson correlation coefficient *r*_pcc_) we refer the reader to the literature,^3,25,55−59^ and the Supporting Information.

## 3 Results and Discussion

### 3.1 Performance for Geometry Optimization

The performance of a given level of theory for geometry optimization is important, because frequencies are obtained in the harmonic approximation, where the molecule must correspond to a minimum on the energy landscape. If the molecular structure is not a minimum, or converges to a different conformation, identified by a high root-mean-square-deviation (RMSD) of atomic coordinates,^50^ imaginary frequencies or incorrect fundamental modes will be observed, respectively. Hence, having a well-defined geometry reference is necessary. It is generally recognized that structure optimizations at the DFT level are much less sensitive to the choice of functional than, for example, energy and property calculations.^60^ For spectroscopic applications with high resolution requirements, modern geometry construction procedures such as templating approaches^61,62^ are available, but are mostly limited to molecules of a few tens of atoms size. Commonly, in case of larger molecules or for “routine” applications, mGGA functionals are sufficient for geometry optimization, where the general recommendation is to use basis sets of triple-ζ quality, or smaller basis sets in combination with corrections for the basis set superposition error (BSSE).^44,60^ Reference geometries for the IR7193 set are available at the B3LYP-3c level of theory. We note at this point that, despite the widespread use of of B3LYP, which is the underlying functional of our reference, some shortcomings for energy and geometry calculations are known.^60,63^ Nonetheless, B3LYP-3c as a hybrid functional with BSSE-corrected basis set, employs corrections for some of those errors (denoted by the “-3c” suffix) and is more than sufficient to compare force-field, SQM, and MLP results. Geometry optimizations for all low-cost methods were herein started from the B3LYP-3c optimized structure in order to ensure convergence to the closest minima on the respective potential energy landscape.

**Figure 2:**
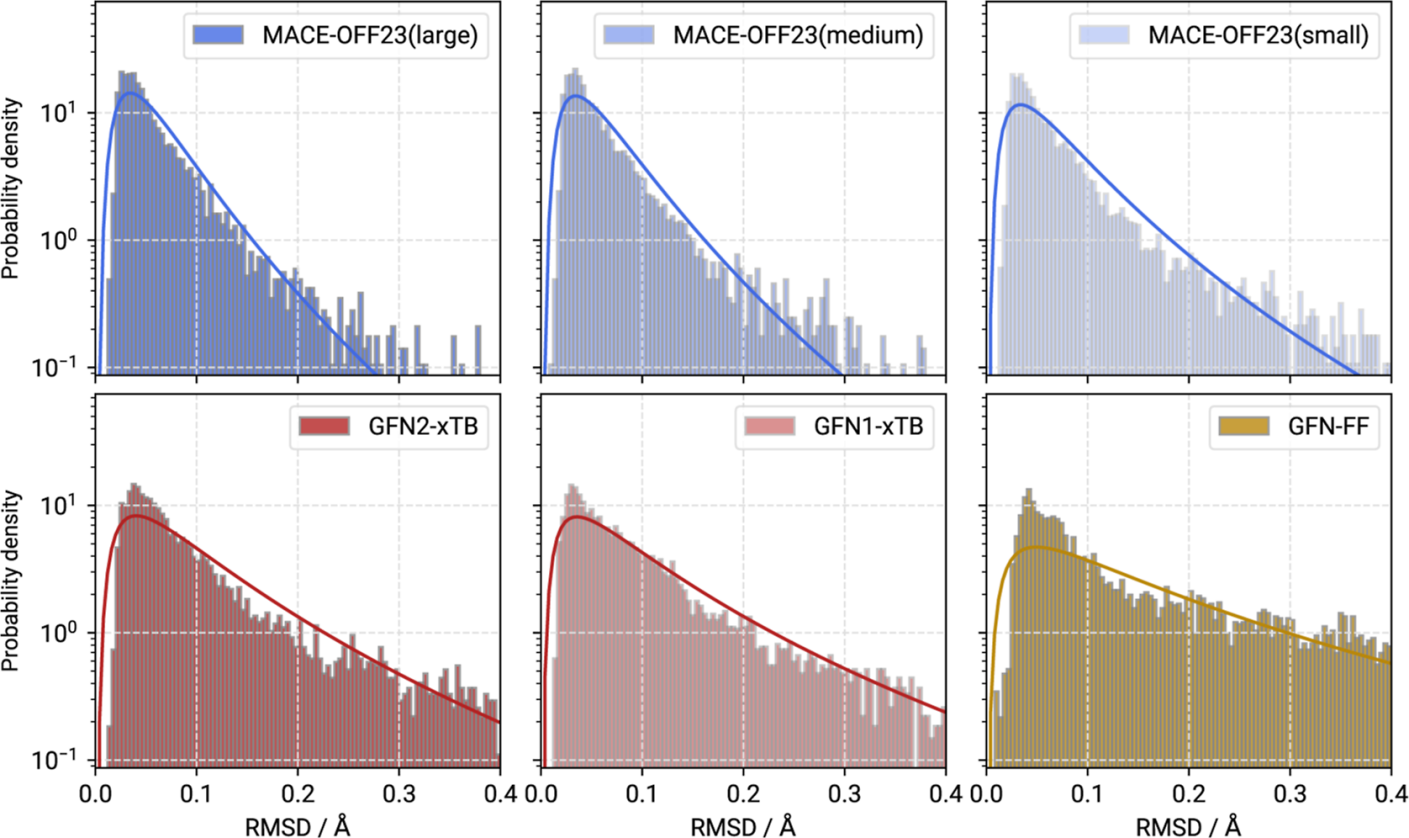
Histograms and fitted log–normal distributions for Cartesian RMSDs calculated between the B3LYP-3c minima and corresponding optimized molecular structures at MACE-OFF23(small/medium/large) and GFN*n*-xTB/FF levels of theory for the IR7193 set. All plots use a logarithmic scale to emphasize the distribution tails.

The GFN1-xTB and GFN2-xTB levels of theory along with the GFN-FF and MACE-OFF23 force-fields were used to optimize and compare structures in the database. Tables 1 and 2 summarize the corresponding RMSD statistics with regard to the B3LYP-3c reference structures. Figure 2 illustrates the distribution of RMSDs as histograms compared to a log–normal distribution. From the presented data we infer that MACE-OFF23 generally seems to outperform the GFN methods for geometry optimization. In Figure 2 this result is shown clearly, where a narrow and sharp log–normal distribution denotes a better performance with regards to the B3LYP-3c reference. Consequently, the average quality of geometry optimization seems to increase in the order GFN-FF, GFN1-xTB and GFN2-xTB, to the MACE-OFF23 models, which is consistent with an intuitive understanding of force-field methods being outperformed by SQM and MLP methods. Within MACE-OFF23, the quality of the optimized geometries increases as one moves from small, to medium, to the large model. The Cartesian RMSD mean is greater than the median because a number of high RMSD outliers are present, which is easily confirmed by the top-heavy log–normal distributions shown in Figure 2. This difference in mean and median decreases with the quality of the MACE-OFF model used. Nonetheless, for all three models the mean and median of RMSDs with reference geometries is below 0.1 Å, which is an excellent performance for this test set. Likewise, over 90% of molecules in the test set have RMSDs below 0.2 Å, illustrating the high probability of any of the three MACE-OFF models in recovering something reasonably close to the DFT geometry. With the exception of the MACE-OFF23(small) model, less than 1% of cases have a high RMSD over 0.5 Å, and only 16 out of 7193 cases exceed an RMSD of 1.0 Å, further attesting to the good quality of the geometries.

**Table 1:** Mean, Median, and Standard Deviation (SD) for Cartesian RMSDs Calculated between the B3LYP-3c Reference and MACE-OFF23(small/Medium/Large) and GFN*n*-xTB/FF Optimized Structures of IR7193^a^

	MACE-OFF23 model			GFN2-xTB	GFN1-xTB	GFN-FF
	small	medium	large			
mean	0.0879	0.0730	0.0694	0.1254	0.1330	0.2339
median	0.0513	0.0478	0.0467	0.0685	0.0733	0.1219
SD	0.1162	0.0873	0.0818	0.1634	0.1852	0.2555

a All values are in Ångström. Narrow distributions indicate better performance.

**Table 2:** Percentage of Structures for IR7193 Falling within the Specified Cartesian RMSD Threshold at a Given Level of Theory

RMSD	MACE-OFF23 model			GFN2-xTB (%)	GFN1-xTB (%)	GFN-FF (%)
[Å]	small (%)	medium (%)	large (%)			
≤0.2	91.51	94.94	95.84	84.75	83.73	61.84
≥0.5	1.63	0.76	0.57	3.95	4.57	14.08
≥1.0	0.22	0.14	0.10	0.56	0.95	1.79

The GFN*n*-xTB methods exhibit excellent performance, although not as good as MACE-OFF23. The mean RMSD of GFN1- and GFN2-xTB slightly exceeds 0.1 Å with 0.1330 and 0.1254 Å, respectively. Still, with over 80% of molecules below an RMSD of 0.2 Å, the SQM methods offer a decent quality for the optimized geometries, although there are significantly more outliers at ≥0.5 and ≥1.0 Å RMSD compared to the MACE MLPs. The classical force-field GFN-FF, with a mean RMSD of 0.2339 Å, is the worst performing method in this test. Only 61.84% of molecules are found below the 0.2 Å mark at this level of theory, which is far less than with the SQM or MLP methods. While clearly having a computational cost advantage compared to the other tested methods, geometries are not as likely to converge to something close to the corresponding DFT minimum.

**Figure 3:**
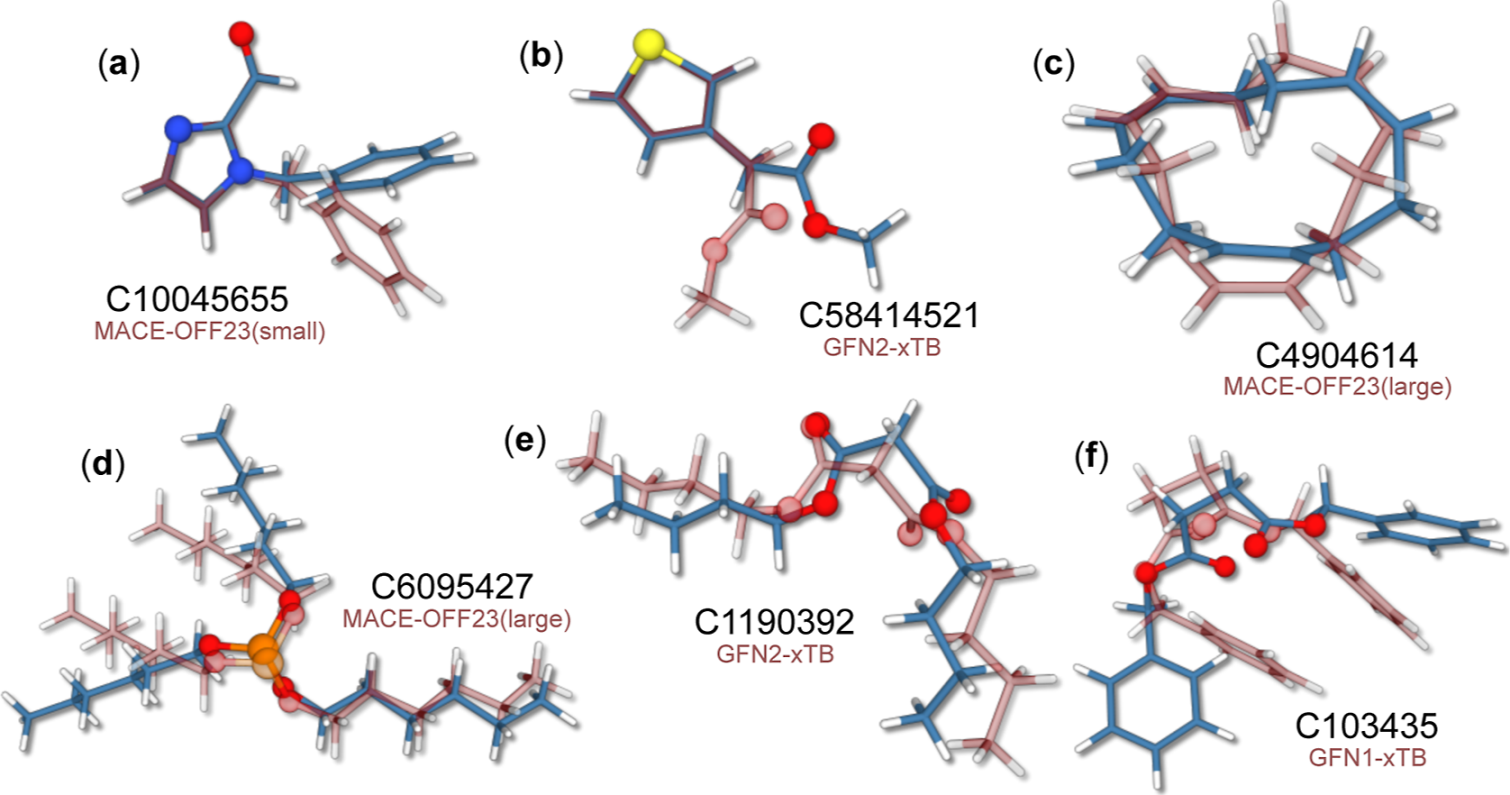
Selected examples for large RMSD cases of molecules from IR7193. Structures shown in blue are the B3LYP-3c reference geometry, structures in transparent red are obtained with low-cost SQM or MLP methods as indicated accordingly. NIST identifiers are given next to each structure. See text for discussion.

Figure 3 showcases some molecules that were not in good agreement with the DFT reference at either the semiempirical or MACE-OFF level of theory. Analysis of the RMSD values from different methods reveals that MACE-OFF generally performed better than the semiempirical methods, with some exceptions. All MLP model sizes and the GFN-FF method incorrectly optimize Figure 3a which shows the MACE-OFF23(small) optimized structure in comparison with the reference. Here, the force-field models seem unable to predict some electronic interactions and MACE-OFF23 and GFN-FF models produce an incorrect orientation for the phenyl group, which hints at a subtle interaction between the aldehyde hydrogen and the phenyl π-system.

The semiempirical methods, on the other hand, account for such effects: the RMSD values for MACE-OFF and GFN-FF models are ∼1.0 Å whereas the xTB Hamiltonians produce the correct conformation and an RMSD of only ∼0.2 Å. Figure 3b shows an overlay of the GFN2-xTB and reference structures. This conformation, starting the geometry optimization from the DFT reference, is selectively produced only by the MACE-OFF23(medium) model and GFN2-xTB. At the B3LYP-3c level, the conformational change is associated with an energy increase by 1.97 kcal mol^–1^, showing it to be a typical asymptomatic case with no clear cause for the mismatch. A similar case is shown in Figure 3c, where the MACE-OFF23(large) conformation is compared with B3LYP-3c. All methods, except for MACE-OFF23(small), have converged to a significantly different structure from the reference with a high RMSD around 1.0 Å. However, the conformation produced by these methods seems to be a more symmetrical minimum, possibly hinting at an incorrect reference structure. This is confirmed by optimization of the B3LYP-3c reference structure at ωB97M-D3(BJ)/def2-TZVPP level, which converges to the same conformation as the low-cost MLP and SQM methods.

Figure 3d, shows another case in which all the low-cost methods disagree with B3LYP-3c. In fact, this molecule, trihexyl phosphite, is the worst-performing case for the MACE-OFF23(large) model, and the only case in which an RMSD of 1.5 Å was exceeded for that level of theory. The conformation produced by all the low-cost methods seems dominated by intramolecular dispersion interactions between two of the three hexyl chains, producing a “hairpin”-like structure, rather than the pinwheel-like reference minimum. Similar to Figure 3c, since all methods disagree with B3LYP-3c, the error could potentially lie with the DFT reference, rather than the low-cost methods. However, the hairpin structure is significantly less stable at the B3LYP-3c level by about 10 kcal mol^–1^. Furthermore, optimization of the B3LYP-3c geometry at the ωB97M-D3(BJ)/def2-TZVPP maintains the same conformation, which suggests that all low-cost methods end up on a different minimum energy path leading to the hairpin minimum during the initial stages of the geometry optimization.

Two less ambiguous cases are shown in Figure 3e,f, which are overlaid by GFN2-xTB and GFN1-xTB minima, respectively. Minima for both molecules are correctly retained by the MACE-OFF23 models. For 3e, the GFN methods inaccurately predict the dihedral angles between the two backbone ester groups, appearing to be a common method error in several of the molecules within IR7193. The presence of long pentyl groups in this molecule leads to high RMSDs of 1.235 to 1.316 Å. For 3f the incorrect geometry is likely a consequence of GFN1-xTB and GFN-FF overestimating the π–π interactions between the two phenyl groups, leading to a “sandwich”-like conformation with an RMSD of ∼1.25 Å for both methods. In summary, while some cases exist that allow a qualitative interpretation of method preference for different conformations, it does not seem possible to quantify model performance for specific intramolecular interactions based on this benchmark set, which needs to be investigated on a by-case basis.

### 3.2 Performance for Vibrational Frequencies

Having discussed performance for optimized geometries in the previous section, we now turn our attention to the calculation of properties defining the IR spectra prediction, with a primary focus on the calculation of internal molecular vibrations in the harmonic approximation. A simple assessment of frequency quality is possible via comparison of the harmonic zero-point vibrational energy (ZPVE), which is approximated^64^ as half the sum over all frequencies
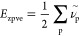
7The ZPVE together with enthalpic and entropic contributions, produces the thermodynamic contributions to the Gibbs free energy in supramolecular calculations.^65^ Although eq 7 is not precise due to neglect of anharmonicity^64^ and a significant amount of information is condensed into the single value of the ZPVE, it offers the distinct advantage of being independent of the permutational order of fundamental frequencies, which can vary with the level of theory used and complicate IR spectra comparison. Furthermore, it allows for the direct derivation of frequency scaling factors, which are commonly applied in (harmonic) DFT frequency calculations to counteract the effects of inadequate descriptions of vibrational anharmonicity.^64,66−69^ Employing an uniform linear frequency scaling factor is the simplest strategy to account for anharmonicity. Depending on the specific application, for example calculation of accurate ZPVEs, or fundamental frequencies in a given frequency range, different scaling factors must be employed.^68−70^ Unsurprisingly, many multiparametric scaling schemes exist that better address some of these issues,^71−75^ such as the “mass-scaling” approach^25,76^ mentioned below. In the present case, we employ the ZPVE as a proxy to determine fundamental frequency linear scaling factors, not to determine good scaling factors for ZPVEs.

The ZPVE, calculated for all molecules in the IR7193 set at the GFN1- and GFN2-xTB, the GFN-FF, and the MACE-OFF23 levels of theory, was evaluated against reference values obtained from B3LYP-3c. To extend the comparison, modified frequencies from a scaled version of B3LYP-3c were used to obtain linear frequency scaling factors, by taking the corresponding fraction of ZPVEs and averaging over the whole test set for each method. The modified B3LYP-3c results, denoted as B3LYP-3c(mscal), employ a special nonlinear scaling scheme following ref (25) which was inspired by the work of Pulay et al.^77,78^ The corresponding “mass-scaling” factors were fitted to the experimental IR spectra and should account for a large part of the vibrational anharmonicity.^25,76^ Using the B3LYP-3c(mscal) as a reference, the ZPVEs were evaluated twice: Once, using the raw ZPVE obtained with each low-cost potential, and once with the ZPVE of each low-cost method based on frequencies scaled by the linear factor determined from the aforementioned fraction. Error measures (mean deviation, mean absolute error, root-mean-square error, and standard deviation) for the investigated levels of theory are shown in Table 3.

**Table 3:** Comparison of Low-Cost Potentials for Calculation of ZPVE^a^

errors	GFN1-xTB	GFN2-xTB	GFN-FF	MACE-OFF23 model		
[kcal mol^–1^]				small	medium	large
Raw Frequencies vs B3LYP-3c Reference						
MD	–2.46	–2.66	–5.40	1.15	1.10	1.09
MAE	2.46	2.66	5.40	1.15	1.10	1.09
RMSE	2.67	2.88	5.78	1.24	1.17	1.17
SD	1.05	1.08	2.06	0.47	0.41	0.40
Raw Frequencies vs B3LYP-3c(mscal) Reference						
MD	1.41	1.21	–1.53	5.02	4.97	4.96
MAE	1.60	1.51	1.57	5.02	4.97	4.96
RMSE	2.19	2.14	1.88	5.54	5.47	5.46
SD	1.68	1.76	1.90	2.34	2.28	2.27
Scaled Frequencies vs B3LYP-3c(mscal) Reference						
scaling factor	0.9913	0.9940	1.0166	0.9602	0.9606	0.9606
MD	0.35	0.48	0.45	0.04	0.03	0.02
MAE	1.01	1.15	1.18	0.23	0.22	0.23
RMSE	1.34	1.57	1.62	0.31	0.30	0.30
SD	1.29	1.49	1.56	0.31	0.30	0.30

a Three sets of data are shown: First values with “raw” B3LYP-3c as a reference, second B3LYP-3c mass-scaled frequencies as a reference (cf. ref (25)), and finally results with linear scaling factors applied and mass-scaled B3LYP-3c as a reference. Values are given in kcal mol^–1^.

When using plain B3LYP-3c as a reference (topmost block in Table 3), the MACE-OFF23 model shows significantly lower errors compared to the GFN methods. Specifically, the MACE-OFF23 model has MAE values ranging from 1.09 to 1.15 kcal mol^–1^ and RMSE values from 1.17 to 1.24 kcal mol^–1^, whereas the GFN methods exhibit much higher errors with MAE values between 2.46 and 5.40 kcal mol^–1^ and RMSE values between 2.88 and 5.78 kcal mol^–1^. However, when mass-scaled B3LYP-3c (considered here as a better “ground truth” for ZPVE calculations, middle block in Table 3) is used as the reference, the performance trend reverses. The GFN methods show improved accuracy, with MAE values between 1.51 and 1.60 kcal mol^–1^ and RMSE values between 1.88 and 2.19 kcal mol^–1^, which are lower than the corresponding errors for the MACE-OFF23 model (MAE: 4.96 to 5.02 kcal mol^–1^, RMSE: 5.46 to 5.54 kcal mol^–1^). GFN2-xTB generally exhibits slightly better accuracy compared to GFN1-xTB, while GFN-FF exhibits the largest errors among the three. Within MACE-OF23, the small, medium, and large models exhibit very similar performance, with only minor variations in the errors, indicating consistent accuracy for harmonic ZPVE calculation by the MLP. These results suggest that the GFN methods represent an adequate off-the-shelf solution for calculation the vibrational frequencies, while MACE-OFF23 exhibits performance closer to the plain hybrid DFT functional an will need accounting for harmonic approximation, for example via rescaling of the corresponding frequencies.

The final block of data in Table 3 provides insights into the linear scaling factors determined using the mass-scaled B3LYP-3c ZPVE as a reference. Notably, the MACE-OFF23 models have consistent scaling factors around 0.96, which is close to the literature scaling factor of 0.97 for B3LYP.^64,68,69^ MAEs for the MACE MLP are lowered to just 0.22 to 0.23 kcal mol^–1^, while RMSEs are lowered to 0.30 to 0.31 kcal mol^–1^. While, to the best of our knowledge, no scaling factors have been investigated for the SPICE ωB97M-D3 reference, the related ωB97X-D3 functional has known scaling factors for fundamental frequencies of around 0.95,^69^ which is close to the observed MACE-OFF23 scaling factors. This result suggests that the MLP behaves similarly to a hybrid DFT method, supporting the previous assessment of its performance. Importantly, a significant harmonic frequency scaling factor shows a systematic error in particular for high-frequency vibrational modes as these dominate the harmonic ZPVE. On the other hand, the GFN methods, being semiempirical and force-field methods, do not exhibit systematic scaling factors, reflecting the less consistent behavior in scaling vibrational frequencies that is known for these levels of theory in the literature.^3,72,79^ Nevertheless, the GFN methods MAE is lowered slightly to 1.01 to 1.18 kcal mol^–1^, and the respective RMSE is lowered to 1.34 to 1.62 kcal mol^–1^. Overall, the consistency in the MACE models underscores their reliability and further validates their use for molecular frequency calculations within the harmonic approximation, aligning closely with hybrid DFT methodologies. Nonetheless, the GFN methods may have an advantage in computational efficiency for drug sized molecules, as addressed in the following.

### 3.3 Computational Efficiency for Harmonic Frequencies

**Figure 4:**
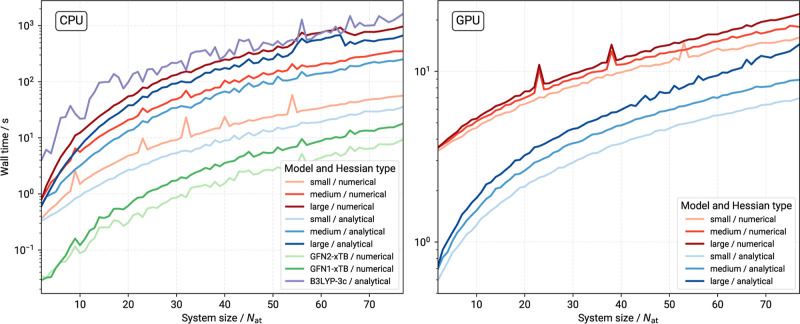
Wall-time comparison for MACE-OFF23 models and Hessian matrix calculations on CPU (left) and GPU (right) devices. Hessian matrices were calculated either by a numerical two-sided difference procedure from the molecular gradients (red), or via auto differentiation (blue). *N*_at_ is the system size (number of atoms). The wall times are given in seconds on a logarithmic scale. All CPU calculations used 4 threads for parallelization and were obtained on a 11th Gen Intel Core i7-11800H (2.30 GHz) processor. The CPU implementation of MACE was used here to ensure direct comparability to the xTB and B3LYP-3c calculations.

Efficiency is a major concern for the high-throughput *in silico* analysis of chemical systems, both in terms of computational cost and in terms of time to solution. In the context of IR spectra, the main computational bottleneck is the computation of the Hessian matrix. For ML potentials, forces are usually obtained via automatic differentiation (autograd). This involves constructing a computational graph of all operations required for the energy calculation and subsequently computing the gradients via backpropagation. Using forces of displaced geometries, (semi)numerical Hessians can be computed, as described above. A second pass through the graph also allows for the computation of second derivatives with respect to the atomic positions, yielding the elements of the Hessian matrix directly. This offers a more efficient and precise way to calculate derivatives, analogous to analytical second derivatives in DFT calculations. Figure 4 shows MACE timings for Hessian calculations using the numerical and analytical implementations as a function of molecule size.

On CPUs (using four shared memory threads), the MACE MLPs are significantly slower than the semiempirical xTB methods for the relatively small molecules investigated herein. For example, the largest molecule in IR7193, with 77 atoms, takes roughly 35 s for a Hessian calculation with the efficient autograd implementation and the smallest, i.e., fastest, MACE-OFF23. The same molecule, using the same computational resources, is processed with a numerical Hessian at the GFN2-xTB level in just 8 s. Similar timings can be achieved, when the MACE models are evaluated using GPU resources, however. On balance, this means that the xTB methods have an edge in terms of computational efficiency in high-throughput applications for drug-like molecules, when only CPU resources are available and xTB is sufficiently accurate.

However, this conclusion is valid only for small systems because the rate-determining step in an xTB calculation formally scales as  (with *N* being the system size with regards to the number of basis functions) which is the cost for diagonalization of the Hamiltonian. Much the same scaling behavior is expected for DFT methods. In the present case, B3LYP-3c exhibits some larger wall time variation with the system size compared to the xTB or MLP methods, which is due to the differently sized basis sets. For example, a molecule with fewer atoms can have more basis functions if it has more heavy atoms compared to a larger system. A prefactor for this scaling comes from the usually high number of self-consistent charge (SCC) iteration cycles required to converge to a solution for large systems. At system sizes of around seven to eight hundred atoms each xTB energy evaluation is roughly the same cost as a MACE-OFF23(medium) evaluation and going beyond that MACE quickly becomes the much cheaper option, even on CPUs. The same “turnover point” for the MACE-OFF23(small) models is located even earlier at around two to three hundred atoms (cf. Supporting Information). Furthermore, xTB may not be able to converge to a suitable SCC solution for large systems at all, experiencing similar self-consistent field issues as a DFT method. On the other hand, no MACE-OFF23(large) calculation was faster than any equivalent xTB evaluation on CPUs. In fact, the MACE-OFF23(large) model calculations on CPUs shows wall times only slightly better B3LYP-3c and for some of the larger molecules requires the autograd implementation to be competitive. B3LYP-3c should here be seen as a baseline for the minimum cost of hybrid DFT methods, as the method employs a rather small basis set, def2-mSVP. Furthermore, the large MACE model has more extensive memory requirements than either the xTB methods or the small and medium MACE models which one needs to consider when performing calculations. In our opinion, the use of the MACE-OFF small and medium MLPs could therefore be beneficial for large amorphous (e.g., peptide and protein) or condensed phase systems, as these models provide the best cost to accuracy ratio. Future work with a thorough investigation of such systems is planned by our groups.

When GPU resources are available, the MACE models are overall the best choice, both in terms of time-to-solution and in terms of accuracy (see below). Notably, the benefit of the autograd implementation is even more significant on GPUs, with autograd Hessians being cheaper for the large model than numerical ones for the small one, across the full investigated size range. Note that for actual application in calculation of IR spectra via the DHA, calculation times are roughly additive. For example, combining a MACE calculation with GFN2-xTB dipole derivatives will require calculation of both the MACE Hessian, as well as the (numerical) dipole derivatives. The latter task requires the same number of function evaluations (6*N*_at_) as the seminumerical Hessian.

### 3.4 Performance for Molecular Dipole Moments

IR intensities are related to the molecular dipole moment μ, as described by eq 5. Therefore, if attempting to switch out the method used for the calculation of ∂μ/∂*Q*_p_ in a composite approach, it is sensible to benchmark the performance for μ. Employing again the IR7193 set of molecules in addition to three conformational benchmark sets (MALT222,^80^ MPCONF196,^81^ and 37conf8^82^), this comparison was done for the B3LYP-3c level of DFT, the semiempirical GFN1- and GFN2-xTB Hamiltonians, the CEH method,^52^ an empirical EEQ model using parameter sets from the D4 dispersion correction and the GFN-FF force-field, as well as a novel MLP for dipole prediction, MACE-μ, and a new fit of the kQEq model^28,29^ for molecules containing the elements HCNOF. Both MACE-μ and kQEq were trained on the SPICE data set^32^ for this study. The ωB97M-D3(BJ)/def2-TZVPPD level of theory was used as a reference for these dipole calculations, and is also used as the reference in the SPICE data set. The ωB97M-D3(BJ) level itself was benchmarked for dipole prediction on a much smaller set of molecules, originally composed by Head-Gordon et al.^83,84^ and refers to CCSD(T)/CBS data, which can be found in the Supporting Information.

**Figure 5:**
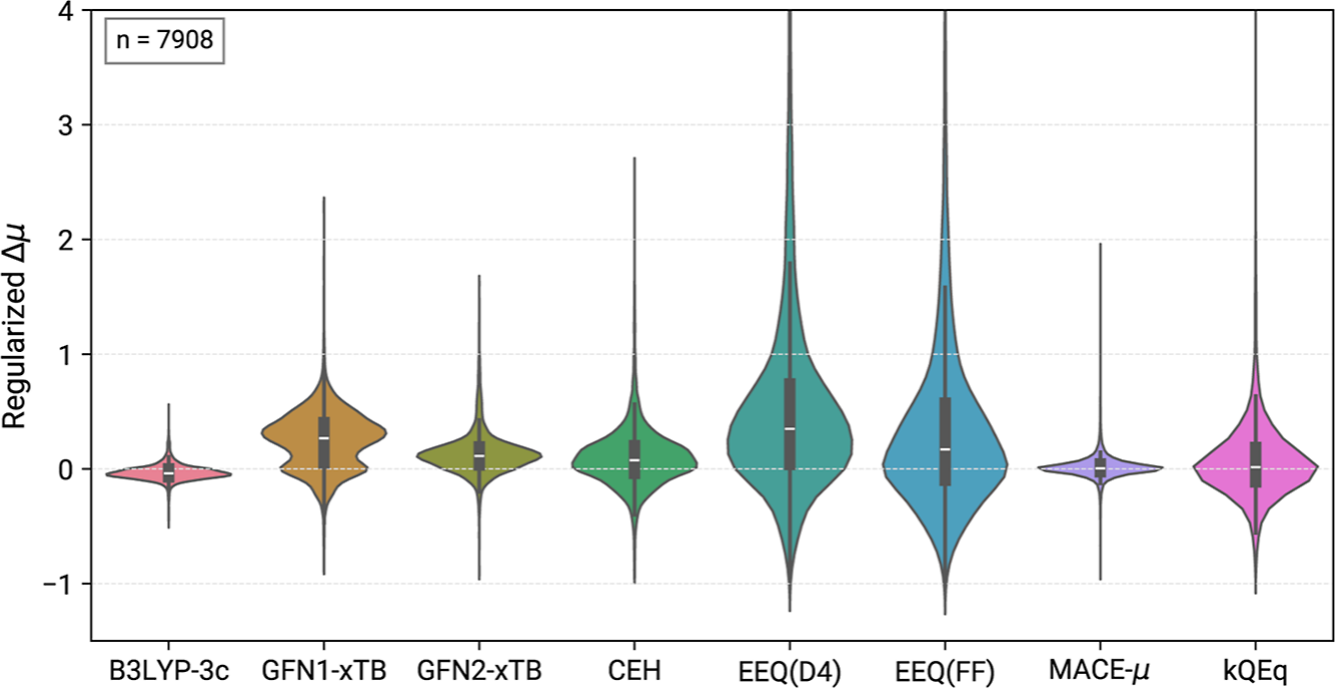
Violin plots for regularized errors of dipole moments at a given level of theory with regard to the reference ωB97M-D3(BJ) dipole moments. Note, kQEq was evaluated for the subset of molecules containing HCNOF elements only.

**Figure 6:**
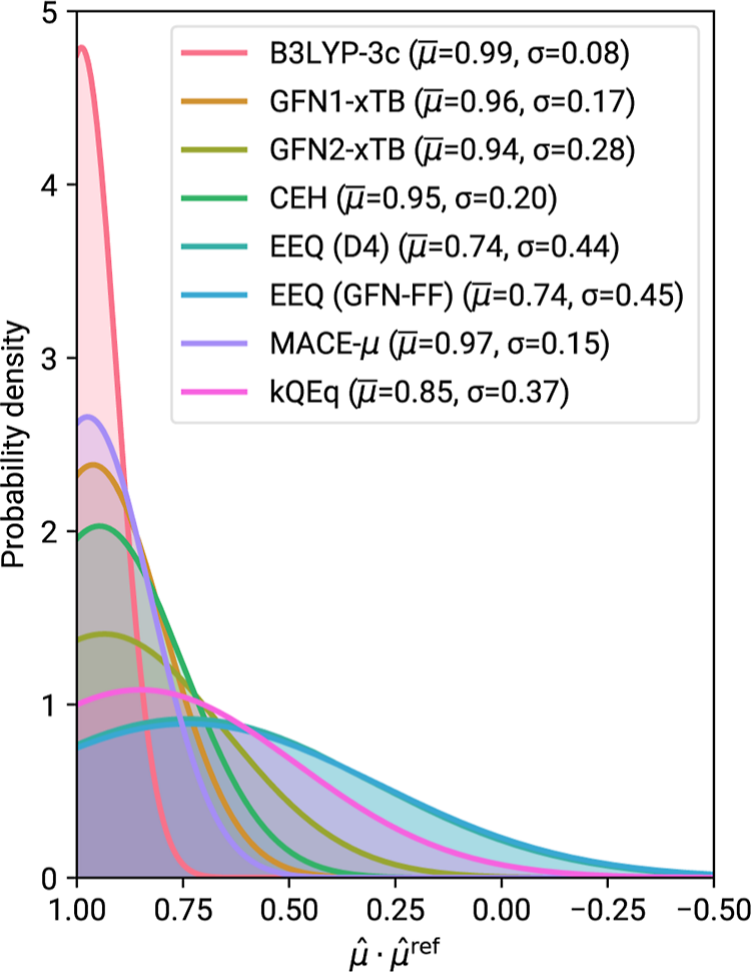
Normal distribution for the dot products of normalized dipole moments at a given level of theory μ̂ and the reference ωB97M-D3(BJ) dipole moments . The respective mean μ̅ and standard deviation σ are given in the legend. Full histograms are reported in the Supporting Information.

Since IR intensities are calculated from derivatives of the dipole moment (eq 6) rather than the dipole moment directly, two criteria are important for the prediction of μ: first, the total dipole moment, and second the orientation of the dipole vector. The total dipole moment is straightforward to benchmark, where we employ the regularized dipole metric Δμ = |μ – μ_ref_|/max{μ_ref_, 1 D} proposed by Hait and Head-Gordon,^83^ in order to handle small and large dipole moments on a more even footing. For reference, typical errors associated with DFT methods are in the range of regularized RMSE’s of 0.05 to 0.10, but larger errors, for example an RMSE of 0.37 for MP2, are not uncommon. While we employ a different reference method, ωB97M-D3(BJ), and a different data set, this level of theory has a rather low regularized RMSE of 0.0475 to 0.0609 on the original benchmark by Hait and Head-Gordon, depending on the basis set (cf. Supporting Information), and hence should provide an appropriate baseline for the low-cost dipole calculations. The corresponding results are shown as violin plots for the summarized IR7193, MALT222, MPCONF196 and 37conf8 sets in Figure 5 and Table 4. Additional information is provided in the Supporting Information. We chose to evaluate the dipole orientation as the dot product of the normalized dipole vectors  of the calculated and reference dipoles, which provides a simple but meaningful way to interpret alignment. Values for this measure can technically be in the range of −1 to 1, where the latter indicates perfect alignment, and values with a negative sign show opposite orientation of the dipole vector. Normal distributions of this measure were fitted for IR7193 and are shown in Figure 6.

**Table 4:** Errors for Regularized Dipole Moments at a Given Level of Theory^a^

	B3LYP-3c	GFN1-xTB	GFN2-xTB	CEH	EEQ(D4)	EEQ(FF)	MACE-μ	kQEq
MD	–0.0325	0.2443	0.1320	0.0941	0.5424	0.3620	0.0128	0.0754
MAD	0.0480	0.1944	0.1213	0.1723	0.5680	0.5451	0.0565	0.2331
RMSE	0.0697	0.2449	0.1877	0.2488	0.8813	0.8351	0.0931	0.4177

a Reference dipole moments were obtained at the ωB97M-D3(BJ) level. Note, kQEq was evaluated for the subset of molecules containing HCNOF elements only. All values are dimensionless.

Several observations can be made for the dipole moment calculations. B3LYP-3c, which serves as our reference for IR spectra, molecular geometries and ZPVE, shows performance very close to the ωB97M-D3(BJ) reference with a regularized RMSE of just 6.97%. Furthermore, as can be seen from Figure 6, B3LYP-3c and the high-level DFT reference also exhibit the best dipole vector alignment with a mean close to unity and a standard deviation of just 0.08. Dipole moments at this level of theory on average seem underestimated, leading to the MD of −3.25%. A probable origin of this error is the small def2-mSVP basis set employed in B3LYP-3c.^84^

GFN1-xTB and the CEH model dipole moments are based on the distribution of Mulliken atomic charges.^48,52^ Unsurprisingly, both methods exhibit similar performance for total dipole moments, with a regularized RMSE of 0.2449 and 0.2488, respectively. Characteristically, GFN1-xTB has two maxima in the violin probability density of Figure 5, resulting in an up-shifted mean compared to CEH and a significant number of molecules have strongly overestimated dipole moments. Both methods contain several outliers, including values overestimated by more than a factor of 2 according to the regularized dipole moments. The alignment of dipole vectors of both methods with the reference is likewise similar, although significantly worse than B3LYP-3c. Overall, GFN1-xTB slightly outperforms CEH. This result can likely be attributed to the SCC procedure in GFN1-xTB allowing a better adaptation of charge density, while CEH is a “single-shot” diagonalization (Hückel) method, lacking self-consistency.

GFN2-xTB improves upon both Mulliken charge based models by atom centered dipole and multipole moments.^49^ While this description leads to a narrower probability distribution and better mean in Figure 5 with a RMSE of 0.1877, dipole alignment is on average worse than with either GFN1-xTB or CEH. Taking this result into account, all SQM methods offer similar robust and efficient dipole moment calculations.

**Figure 7:**
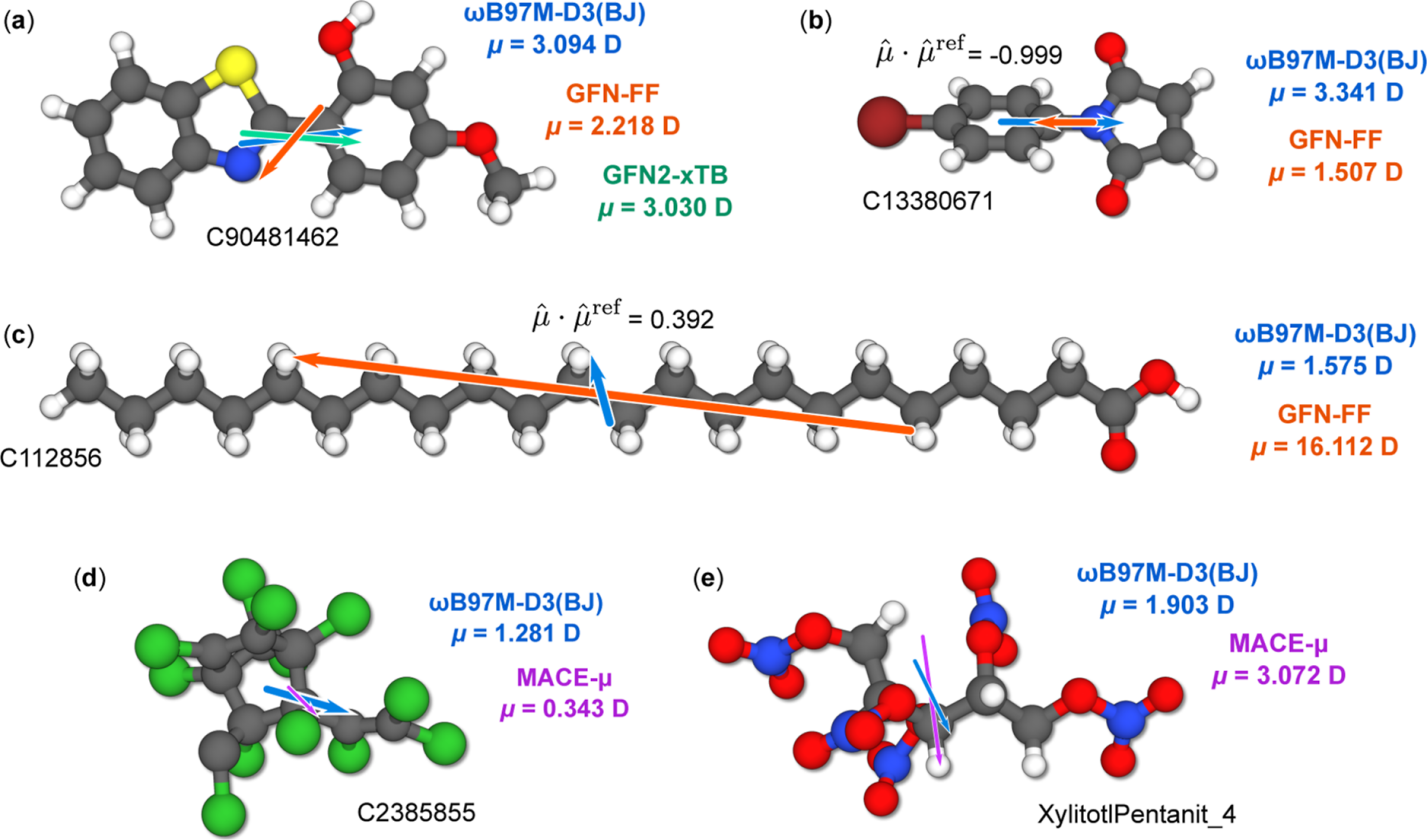
Example molecules with depicted dipole moments. (a) Benzothiazolyl methoxyphenol (NIST Id C90481462), (b) Bromophenyl maleimide (NIST Id C13380671), (c) Docosanoic acid (NIST Id C112856), (d) “mirex” (NIST Id C2385855), and (e) Xylitotl pentanit from the 37conf8 benchmark set. Dipole moments are depicted to-scale as colored arrows, where blue refers to ωB97M-D3(BJ), red-orange refers to EEQ(GFN-FF), green refers to GFN2-xTB and margenta refers to MACE-μ(medium). Debye values are also included. Figures (b,c) furthermore illustrate the alignment measure  for the two depicted levels of theory.

MACE-μ, a MACE model trained on the SPICE^32^ data set to predict the molecular dipole vector for this work, shows some interesting features. The model presented here formally refers to a “medium” MACE model size, some additional data for a “small” model can be found in the Supporting Information. The MD of 0.0128, the MAD of 0.0565 and RMSD of 0.0931, as well as the dipole vector alignment (cf. Figure 6), show a generally better performance of MACE-μ than those of the SQM-based methods. Instead, the MACE-μ performance much more closely resembles that of a DFT method, although some greater outliers can be seen in the violin plots of Figure 5. Notably, our dipole moment reference method is the same as the level of theory employed in the SPICE data set and, in fact, a number of predictions match the ωB97M-D3 reference precisely. Upon visual inspection of structures exhibiting regularized dipole moment errors approximately double the RMSE, we identified several limitations of the MACE-μ model. Notably, structures containing multiple halides, such as chlorine and bromine, tend to be represented poorly. Similarly, structures with high concentrations of nitrate and hydroxyl groups frequently appear among failure cases, potentially indicating a pattern of unreliability. Two examples are shown in Figure 7 below. An additional pattern emerges with molecules containing sulfur and phosphorus: phosphorus is commonly found with high regularized errors in abundance even among the outliers, whereas sulfur-containing systems are associated with high regularized errors, but overall less so than the phosphorus systems. This observation suggests that these failures are systematic for heavier elements. The MACE-μ models may struggle with specific chemical motifs that are not well represented in the SPICE training data, warranting further investigation, or possibly retraining to enhance robust performance across a wider range of molecular systems. At the present time it is unclear what causes the remaining dipole moment outliers of MACE-μ, but still we see it as a slightly better (although for small systems more expensive) alternative to the GFN*n*-xTB dipole predictions.

The worst molecular dipole moments come from the classical EEQ models. The RMSE, 0.8812 and 0.8351 for the D4 and GFN-FF parameter set, respectively, is more than an order of magnitude worse than for B3LYP-3c, and roughly a factor of 4 worse than with the SQM methods. This is in line with previous observations for dipole moments calculated from EEQ models in the literature.^25,28^ The performance can apparently be influenced by the parametrization, and the GFN-FF EEQ model, on average, shows marginally better results than the D4 EEQ parameter set. Unsurprisingly, the largest outlier, with a dipole moment overestimated by roughly a factor 10, is obtained with EEQ(D4). For the dipole vector orientation both EEQ parameter sets perform equally badly, giving broad distributions in Figure 6 that extend even into the negative regime of . Hence, a non-negligible number of molecules have dipole moments pointing into the wrong direction at this level of theory, two of which are shown in Figure 7a,b. In these cases, EEQ models clearly provide an insufficient description of the charge distribution, which can only be recovered with electronic structure calculations or by certain MLPs.^28^ EEQ models are prone to unphysical charge-transfer problems,^17,28,85^ which contributes to these issues.

Interestingly, the kQEq model (which is an ML augmented EEQ model) performs significantly better than its classical counterparts. In terms of the dipole moments it approaches the accuracy of GFN1-xTB, although several significant outliers lead to a somewhat larger RMSE. The dipole orientations are also improved relative to the classical models, although a few outliers with dipoles pointing in the wrong direction remain. Overall, environment dependent electronegativities, as used in kQEq, improve the performance of EEQ-based models, but the underlying limitations (i.e., using a point-charge representation of the density and unphysical charge transfer effects of EEQ) are not fully cured.

Figure 7c shows the aforementioned high EEQ dipole moment outlier. A similar linear molecule has been discussed, for example, in ref (28). Interestingly, this outlier cannot be attributed to the partial charge distribution of the carbon atoms, as illustrated by Figure S3 in the Supporting Information. In fact, all atomic charges at the EEQ(GFN-FF) level are very similar to those at the GFN2-xTB level, despite the latter calculation exhibiting a qualitatively well-defined dipole moment that differs from the high-level DFT reference by just 0.204 D (or 13%, referring to the regularized dipole measure). On the other hand, partial charges at the EEQ(D4) level are different from both GFN2-xTB and EEQ(GFN-FF), as well as the DFT references. A clearly wrong assignment of charges that would be reflected in the dipole moment prediction cannot be discerned in either case, although partial charges calculated at the high quality reference ωB97M-D3(BJ) differ distinctively from all other methods. The severely overestimated dipole moments via the EEQ models can therefore not be unequivocally attributed to charge transfer issues from polarization or electron delocalization. A delicate balance between different contributions to the molecular dipole moment is needed for useful performance.Two examples of high MACE-μ errors are shown in Figure 7d,e. While the dipole orientations are not very different, the absolute dipole moment is strongly underestimated in the first molecule, and overestimated in the second. As stated above, these errors seem to occur primarily for systems with compositions not well represented in the SPICE training data set, which is expected. However, upon closer inspection we see that the “mirex” structure of Figure 7d is actually chemically incorrect and features a broken C–C bond. This chemical error is present already in the NIST database reference conformer and while it does not significantly affect theory-to-theory comparisons, a comparison to experimental data would be flawed. A small percentage of NIST entries seem to feature such errors. More information is provided in the Supporting Information.

### 3.5 Performance for Simulation of Gas-Phase IR Spectra

Finally, combining predictions of harmonic frequencies and Cartesian dipole derivatives via eq 6, allows the prediction of IR spectra. In principle, any combination of methods for frequencies and dipole moments can be used in this approach. However, both quantities are required for the same stationary point, meaning that dipole moment derivatives in particular must be obtained for molecular structures optimized at the same level of theory that is employed for the frequency calculation.

**Figure 8:**
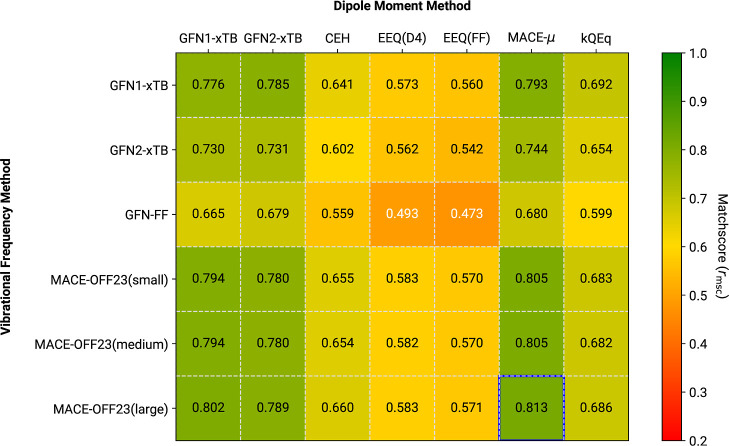
Average *r*_msc_ comparison for different frequency/dipole method combinations. Reference data refers to “raw” B3LYP-3c IR spectra of the IR7193 set. Neither the tested methods, nor the reference, employ any kind of scaling to the harmonic frequencies. The overall highest average matchscore is marked by a blue outline.

**Figure 9:**
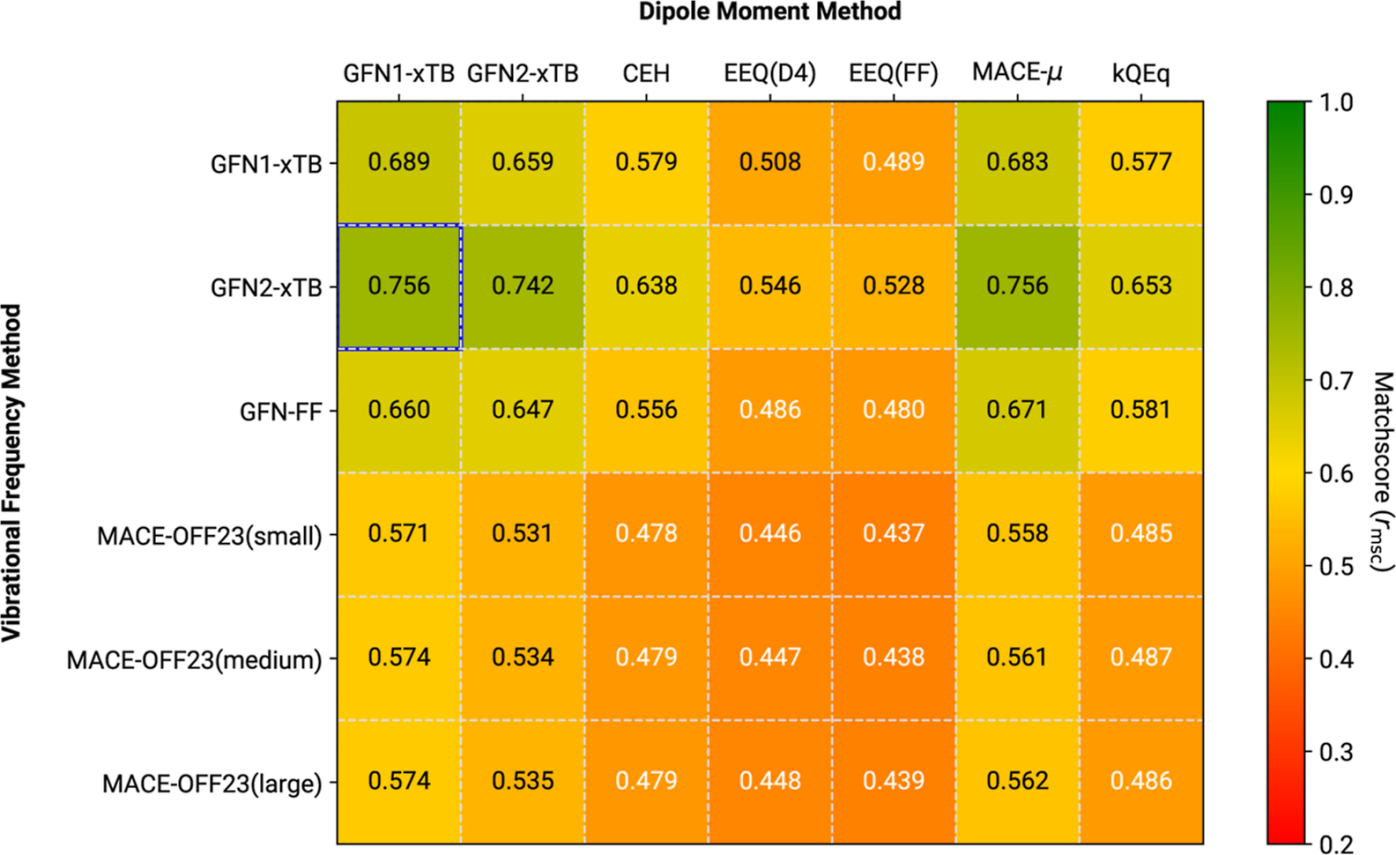
Average *r*_msc_ comparison for different frequency/dipole method combinations. Reference data refers to mass-scaled B3LYP-3c IR spectra of the IR7193 set. Neither of the tested methods employs any kind of scaling to the harmonic frequencies. The overall highest average matchscore is marked by a blue outline.

**Figure 10:**
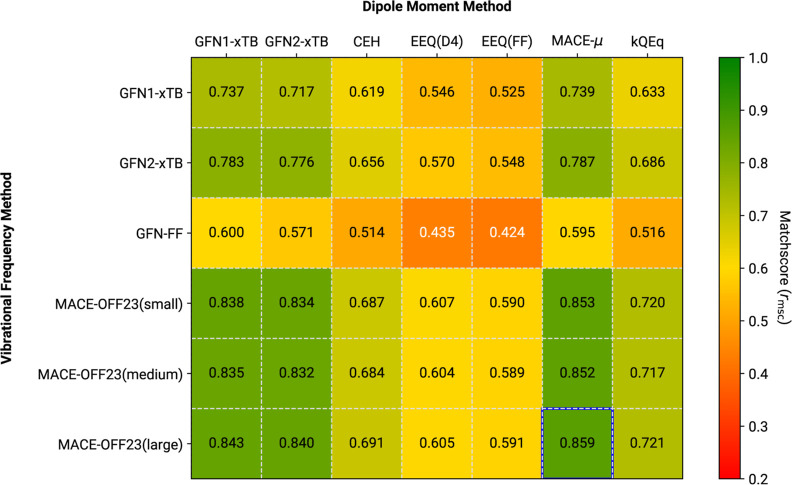
Average *r*_msc_ comparison for different frequency/dipole method combinations. Reference data refers to mass-scaled B3LYP-3c IR spectra of the IR7193 set. Harmonic frequencies from the low-cost methods were scaled by the factors from Table 3. The overall highest average matchscore is marked by a blue outline.

For the IR7193 set, we investigated all combinations of the discussed levels of theory. Two sets of reference data were employed: unscaled “raw” B3LYP-3c and the respective mass-scaled results, which closely correspond to experimental data.^25^ For brevity, the following discussion focuses on the *r*_msc_ matchscore metric, with other metrics provided in the Supporting Information. Results are summarized in Figures 8–10, illustrating the average *r*_msc_ over IR7193 for all method combinations, referencing different frequency scaling for either reference or actual data. Further results for comparison with experimental rather than B3LYP-3c spectra can be found in the Supporting Information and are explicitly omitted here due to the aforementioned conformational and measurement effects. Figure 8 presents the average *r*_msc_ with respect to unscaled B3LYP-3c reference spectra. As expected, there is a correlation between previously observed performance for frequencies, dipole moment prediction, and the similarity scores for IR spectra. All MACE-OFF23 variants consistently outperform the GFN*n*-xTB methods, regardless of the origin of the dipole data employed. The best average *r*_msc_, with a value of 0.813, is provided by MACE-OFF23(large) in combination with MACE-μ based IR intensities. Combinations of MACE-OFF23 with dipole derivatives obtained from either of the xTB methods also show high *r*_msc_ values, around 0.79. Unfortunately, IR intensities obtained via the classical EEQ model show much poorer performance, reflecting the previously observed lack of accuracy in dipole moment prediction. The semiclassical CEH model performs between the xTB and EEQ-derived IR predictions, contrary to the similar behavior of this method for dipole moments compared to GFN1-xTB (see Section 3.4). A comparable performance, moderately improving on CEH, is observed for IR spectra with intensities predicted by the kQEq method. As observed for the dipole moments above, kQEq significantly improves upon the classical EEQ models, but the underlying methodical limitations prevent reaching the accuracy of a self-consistent semiempirical treatment. However, note again that kEQq was trained only for the HCNOF element subset and matchscore averages are not fully comparable to the other methods. In general, similar trends are observed for the combination of GFN*n*-xTB frequencies with various dipole moment methods: MACE-μ and xTB-derived IR intensities are accurate, while classical and CEH-based IR intensities are not. Employing a force-field, even one trained for frequency prediction such as GFN-FF, notably worsens the obtained IR spectra. Multiple factors probably contribute to this performance. Most importantly, GFN-FF has a significant number of large RMSD minima after optimization, as discussed in Section 3.1, which naturally affects the predicted frequencies and dipoles and hence must contribute to the poor results. The best predictions are again obtained in combination with GFN*n*-xTB or MACE-μ calculated IR intensities, although the associated computational costs exceed those for calculating the GFN-FF Hessian.

Employing mass-scaled reference data to better reproduce experimental gas-phase spectra^25^ has a significant impact on the observed performance, as shown in Figure 9. All MACE-OFF23-based calculations show substantially poorer *r*_msc_ values, regardless of the employed dipole moment methods, which can be clearly assigned to the shift in frequencies. On the other hand, all GFN methods are much less affected by the changing reference. GFN2-xTB performs better than its predecessor GFN1-xTB, and even has slightly higher *r*_msc_ than with the plain B3LYP-3c reference. Apparently, the formulation of the GFN method family is more consistent in reproducing the molecular frequencies (as observed already for the ZPVE, see Section 3.2), and hence preform similarly in both cases. This result shows that the GFN methods are a reasonable choice for inexpensively calculating frequencies, since, on average, robust performance is expected. Unfortunately, this observation also implies a general inability of the GFN methods to achieve much better IR spectra predictions, as well as potential shortcomings of the comparison metric, which is discussed below.

In Figure 10 both the reference data and the low-cost simulations target IR spectra closely corresponding to the experiment.^25^ Compared to Figure 9, the behavior of MACE-OFF23-based predictions with regard to the mass-scaled B3LYP-3c reference can be rectified by employing the linear frequency scaling factors obtained in Section 3.2. In combination with either the xTB-based dipole derivatives or MACE-μ, all MACE-OFF23 MLPs consistently achieve average *r*_msc_ over 0.8, with the overall best benchmark of 0.859 for MACE-OFF23(large) + MACE-μ. A similar trend is seen for GFN1-xTB and GFN2-xTB, with the latter theory performing better. The best average *r*_msc_ among these SQM methods is 0.787, which is achieved with GFN2-xTB in combination with MACE-μ. Combining the MACE-OFF23 frequencies with kQEq-based IR intensities generally passes a *r*_msc_ of 0.71, which makes them roughly comparable to GFN1-xTB IR spectra employing GFN2-xTB dipole derivatives, moderately improving upon the CEH results. As before, GFN-FF-based predictions of IR spectra, as well as all IR spectra calculated with underlying EEQ or CEH dipole moment derivatives, are lacking accuracy. In fact, GFN-FF results scaled by the linear factor determined via the ZPVE are on average worse than both these alternatives, as seen in Figures 8 and 9, which is further testimony to an asystematic behavior of the method. For the underlying dipole moment derivatives, classical charge-equilibrium models exhibit relatively poor average *r*_msc_ values throughout, independent of both reference values and methods used for frequency calculation. Hence, their use in IR spectra computation should generally be avoided. The semiclassical CEH model, on the other hand, while certainly worse than either a SQM or specially trained MLP, shows promising performance. Results for this method could certainly be improved with a specialized parametrization, and motivate further development of similar single-diagonalization SQM Hamiltonians.

The overall best performing DHA combination, MACE-OFF23(large) + MACE-μ, was further investigated to outline systematic shortcomings of the simulated spectra. For this purpose, the 170 lowest matchscore cases, i.e., all those showing a *r*_msc_ below an arbitrarily chosen cutoff of 0.7, were visually inspected. As previously seen from inspection of the MACE-μ results in Section 3.4, low matchscore cases seemingly correspond to chemistry that is not well represented in the SPICE training data set, and a few motifs occur often in the 170 molecules. Specifically, sulfur containing systems make up 27.7% of these cases, most of which have isothiocyanate groups present. Other common motifs occurring multiple times are isonitrile functional groups, oxime groups, and stick-like molecules containing alkyne triple bonds. Surprisingly, phosphorus is present only four times in the inspected spectra, despite being identified as one of the main error sources in the MACE-μ results above. Far larger errors are caused by small systems with two to six atoms, and small systems with multiple heavy, in particular halogen, atoms. While the latter effect must be attributed again to the SPICE set composition as training foundation, errors for the smallest systems stem from the DHA methodology. For example, the system with the overall smallest *r*_msc_ of 0.234 is CO_2_. The corresponding spectrum only has two IR bands, namely the bending and the asymmetric stretching mode. While relative intensities for the two bands are predicted sufficiently well, the peak positions are shifted compared to the reference, leading to low overlap in the matchscore calculation. Small systems with a few sharp IR bands are prone to such errors, even for modest shifts.

**Figure 11:**
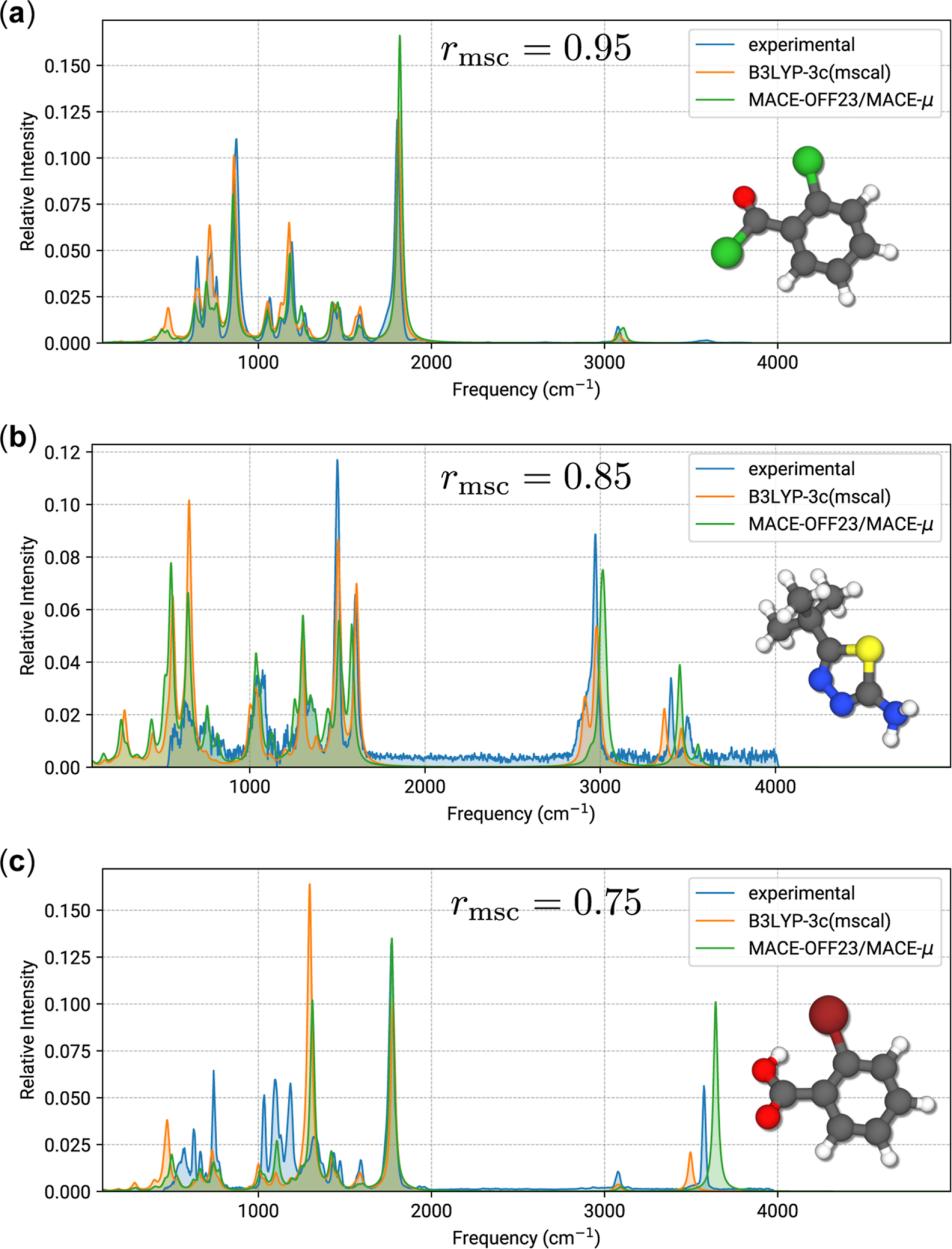
Examples for typical matchscore regimes. (a) A “good” matchscore case of *r*_msc_ = 0.95 (NIST Id C609654), (b) an “average” matchscore case of *r*_msc_ = 0.85 (NIST Id C39222736), (c) a “mediocre” matchscore case of *r*_msc_ = 0.75 (NIST Id C88653). Computed MACE-OFF23(large)/MACE-μ spectra have frequencies scaled by a factor of 0.9606. *r*_msc_ refers to the comparison between B3LYP-3c and MACE.

Unfortunately, these results can also be interpreted as a lack of precision in matchscore-based metrics like *r*_msc_. While clearly defined or linearly shifted spectra are sufficiently well compared by matchscore metrics, for asystematically behaving methods the comparison is too insensitive to accurately distinguish spectra, and will produce some average *r*_msc_ value (as seen for GFN1-xTB and GFN2-xTB, as well as their combination with MACE-μ in Figure 9). A better metric should include clear assignment of vibrational modes between reference and calculated spectra, for which, to the best of our knowledge, no automated procedure exists, although attempts exist to project the IR intensity along localized modes and use an appropriate assignment accordingly.^86^ Furthermore, such a metric would only work if it is known a priori that the same conformation of the same molecule is to be compared and information about the vibrational modes is accessible for the reference spectra. If the goal is, for example, the identification of unknown compounds from experimental measurements, only matchscore metrics seem suitable. Hence, due to lack of better alternatives, we continue to refer to *r*_msc_, but advise caution to the reader. On the other hand, obtaining generally mediocre values of *r*_msc_ as seen for GFN*n*-xTB, may signify suitability in applications where an exact order of the frequencies and modes is not overly important, for example the calculation of the ZPVE or vibrational entropy. This fitness is in line with the original GFN*n*-xTB design purpose^17^ and hence seems to be confirmed by our results. For a visual assessment of *r*_msc_, examples of a “good”, “average” and “mediocre” matchscore are given in Figure 11, depicted as an overlay between the mass-scaled B3LYP-3c reference and the MACE-OFF23(large)/MACE-μ computed IR spectra with applied ZPVE scaling factor. Additionally, experimental spectra from the NIST database are included but no matchscores are provided. In general, matchscores of 0.9 or higher exhibit a close match between the two compared spectra, with only minor differences in relative intensities. For Figure 11a, this quality of agreement is seen between both calculated spectra and the experimental gas-phase IR spectrum. Scores between 0.8 and 0.9 likewise denote good correspondence of spectra, although some frequency shifted signals may appear, as seen for modes around 3000 to 3500 cm^–1^ in Figure 11b. In this case, the experimental spectrum is noisy, has several overlapping bands, and is missing the low-frequency fingerprint regime, which leads to a visually worse agreement. The noisiness of the experimental spectrum clearly impairs the calculation of any comparison metric and thus demonstrates the benefit of theory-to-theory comparisons. Spectra with *r*_msc_ below 0.8 commonly disagree with regard to at least one signal, as for example the OH vibrational mode at around 3500 cm^–1^ in Figure 11c, where both the frequency and intensity are overestimated by the low-cost computation. A mismatch of this type is a common cause of “mediocre” or worse matchscore results, despite other regions of the spectrum (e.g., the fingerprint regime up to ≈1500 cm^–1^) exhibiting good agreement. Neither of the two calculated spectra agrees very well with the experimental spectrum, in particular because bands in the fingerprint regime underestimate intensities or are entirely absent. The OH mode is found experimentally in between the DFT and MACE-predicted signals. Since both calculations refer to the same conformation and conformational effects can be ruled out, it is likely that the mere presence of bromine and typical challenges associated with heavy atoms are the cause for limited accuracy in the predictions. Specifically, it is reasonable to assume some anharmonicity due to the heavy atom that is not well captured by the scaled DHA, that the small basis of B3LYP-3c is not sufficient to describe bromine, and that bromine is too sparsely represented in the SPICE data set used in the MACE fits. These are typical issues in the presented DHA framework warranting awareness.

Finally, spectra with overall fewer IR signals are more prone to errors due to shifted IR bands, while signals in cluttered spectra will often overlap regardless and hence contribute to *r*_msc_. Match scores can be further artificially inflated by choosing too large fwhm parameters for line broadening, as discussed by Henschel et al.^3^ A similar effect arises from the spectral complexity: Overall more IR bands will convolute a complex spectrum, thus increasing the possibility to obtain higher overlap within the matchscore metric and to observe false-positive band assignments. Since spectral complexity increases mainly with the system size, caution is be advised for large molecular systems. Here, the application of similarity metrics to identify spectra may not be feasible. For benchmark purposes with theory-to-theory comparisons being made, a new metric depending on identification and matching of the actual vibrational mode would clearly be preferable.

## 4 Conclusion

In this work, we tested combinations of low-cost semiempirical, force-field, and MLP methods for calculating gas-phase IR spectra within the double-harmonic approximation. Our approach involves benchmarking the GFN1-xTB, GFN2-xTB, GFN-FF and MACE-OFF23 models for calculating molecular geometries and associated harmonic frequencies, as well as the GFN1-xTB, GFN2-xTB, CEH, EEQ and MACE-μ models for molecular dipole moments. Our analysis focuses mostly on the IR7193 set, collecting some 7193 organic molecules and their IR spectra. Linear frequency scaling factors were determined based on the ZPVE fraction relative to a specially (mass-)scaled theoretical reference, B3LYP-3c. IR spectra were obtained by combining frequency predictions with IR intensities calculated via numerical dipole moment derivatives.

A key observation is that the pretrained MACE-OFF23 MLPs are found to be particularly effective for drug-like molecules, providing accurate geometries, frequencies, and IR spectra that outperform the GFN*n*-xTB methods. Interestingly, MACE-OFF23 appears to inherit characteristics similar to DFT methods, as evidenced by the comparable importance and magnitude of harmonic frequency scaling factors. This result contrasts with the evaluated semiempirical methods, which are mostly asystematic in regard to such treatments, and on average are only little affected by a linear scaling of the harmonic frequencies. In general, although the GFN*n*-xTB methods offer decent performance and speed for smaller system sizes and inherently include dipole moments, they often do not match the accuracy of MACE-OFF23. This result is clearly seen from the best observed average *r*_msc_ of 0.787 for GFN2-xTB versus a *r*_msc_ of 0.859 for MACE-OFF23(large), both in combination with MACE-μ-based IR intensities. The smaller and therefore faster MACE-OFF23(small) and MACE-OFF23(medium) models offer comparable efficacy to their large counterpart. Both MACE-μ and GFN*n*-xTB exhibit reasonable performance for calculation of the underlying dipole moment derivatives, with the SQM methods having a computational cost advantage for drug-sized molecules. The use of classical EEQ models for this purpose should be avoided. While CEH does not reach the accuracy of SQM or MLP dipole predictions, the observed performance is a clear motivator for future research.

In summary, we see an opportunity for both MLP and SQM-based prediction of IR vibrational spectra, or a combination of techniques. MACE-OFF23, in particular, provides excellent performance for molecular geometries and frequencies, making it a promising method for calculating thermostatistical contributions for large systems in addition to the spectroscopic applications. For very large systems, exceeding roughly 700 atoms, we expect MACE-OFF23 to gain a computational cost advantage over the xTB methods due to the MLPs better scaling behavior with system size. Future work should focus on investigating more accurate reference frequencies both by going beyond the B3LYP-3c level of theory, and extending beyond the harmonic approximation to include vibrational anharmonicity. Investigating IR spectra for condensed-phase and large amorphous systems, such as proteins, using MACE-OFF23, is another promising avenue of research. New MLP developments, especially the inclusion of proper electrostatics,^87^ will be beneficial for this purpose and could allow IR intensity prediction without the need for separate dipole models. Additionally, exploring other spectroscopic techniques, such as Raman spectroscopy by examining polarizability instead of dipole moments, will be valuable.

## Data Availability

Additional raw data can be provided by the authors upon request.

## Supporting Information Available

Several spreadsheets with calculated comparison metrics are provided (ZIP)
Additional data and discussion related to the manuscript, in particular additional dipole benchmark evaluation and comparisons of experimental IR spectra with the calculated data. Furthermore, input geometries (PDF)

## Abbreviations

BSSE: basis set superposition error
B3LYP-3c: short notation for B3LYP-D3(BJ)^ATM^-gCP/def2-mSVP
CEH: charge extended Hückel model
CN: coordination number
CREST: Conformer-Rotamer Ensemble Sampling Tool
DFT: density functional theory
DHA: double-harmonic approximation
EEQ: charge equilibrium model
FF: force-field
GFN: geometries, frequencies, noncovalent interactions
GFN-FF: GFN force-field
IR: infrared (spectroscopy)
kQEq: kernel charge equilibration model
MACE: higher order message passing atomic cluster expansion
MACE-OFF23: MACE organic force-field
MACE-μ: MACE dipole model
MAD: mean absolute deviation
MAE: mean absolute error
MD: mean deviation
ML: machine learning
MLP: machine learning potential
PEL: potential energy landscape (synonym to PES)
PES: potential energy surface (synonym to PEL)
RMSD: root-mean-square-deviation (of atomic positions)
RMSE: root-mean-square-error
SPICE: Small-molecule/Protein Interaction Chemical Energies data set
SQM: semiempirical quantum mechanics
xTB: eXtended Tight-Binding
ZPVE: zero-point vibrational energy
