# Supplementary material for: Efficient Composite Infrared Spectroscopy: Combining the Double-Harmonic Approximation with Machine Learning Potentials
Source: J Chem Theory Comput. 2024 Dec 12;20(24):10986–1004. doi: 10.1021/acs.jctc.4c01157 (PMC11672665; doi:10.1021/acs.jctc.4c01157)
Supplement: Supplementary file 2 — ct4c01157_si_002.pdf [file ct4c01157_si_002.pdf]

Supporting Information: Efficient Composite Infrared Spectroscopy: Combining the Double-Harmonic Approximation with Machine Learning Potentials

Philipp Pracht,[*,†,‡] Yuthika Pillai,[†] Venkat Kapil,[¶,†,§] Gábor Csányi,[‖] Nils Gönnheimer,[⊥] Martin Vondrák,[⊥] Johannes T. Margraf,[⊥] and David J. Wales[†]

†*Yusuf Hamied Department of Chemistry, University of Cambridge, Lensfield Road, Cambridge CB2 1EW, UK*

‡*Department of Chemical Engineering, Massachusetts Institute of Technology, Cambridge, Massachusetts 02139, United States*

¶*Deptartment of Physics and Astronomy, University College London, 17-19 Gordon St, London WC1H 0AH, UK*

§*Thomas Young Centre & London Centre for Nanotechnology, 19 Gordon St, London WC1H 0AH, UK*

‖*Engineering Laboratory, University of Cambridge, Trumpington Street, Cambridge CB2 1PZ, UK*

⊥*University of Bayreuth, Bavarian Center for Battery Technology (BayBatt), 95448 Bayreuth, Germany*

E-mail: research@philipp-pracht.de

1 Calculation of spectra comparison scores

In computed spectra the frequencies and intensities of the vibrational modes are available as isolated signals ('*stick spectrum*') and for detailed comparisons have to be expanded into the same spectral domain as, e.g. experimental data. This is achieved by employing a Lorentzian line shape function for each mode

$$\phi_p(\nu) = I_p \left(1 + \frac{\nu_p - \nu}{0.5w}\right)^{-1} \quad , \tag{S1}$$

where ν_p is the position (calculated frequency) of the mode p, I_p is its intensity and w is the full width at half maximum (FWHM). Typical values employed for the FWHM in range from 20 to 40 cm^{-1}, whereas the average line width in experimental spectra was determined as 24 cm^{-1}.[S1] The simulated spectrum is then simply given by the sum of all the Lorentzian functions over all N_p modes

$$\Phi_{\mathrm{norm}}(\nu) = I_{\mathrm{norm}} \sum_p^{N_p} \phi_p(\nu) \ , \tag{S2}$$

with the normalization constant I_{norm}. Such spectra are normalized by $\sqrt{\int \Phi_{\mathrm{norm}} \mathrm{d}\nu} \overset{!}{=} 1$ and in this form are directly (point wise) comparable to experimental and other computed data. Normalizations to single signals, e.g., the largest peak of the spectrum, should be avoided because relative intensities and frequencies are strongly dependent on the theoretical level and hence it cannot always be ensured that the same peak is selected. Finally, from the two normalized spectra that shall be compared, two k-dimensional vectors (u and v) are constructed where u_i is the normalized intensity of the i-th point in the spectrum and du is a predefined distance resolution between the points u_i and u_{i+1}. In summary, the FWHM, k and du are input parameters affecting the comparison. For consistency, all comparisons employed the same defaults with du set to 1.0 cm^{-1}), and k chosen accordingly in the regime between 100 and 4500 cm^{-1}. For the FWHM a value of 30 cm^{-1} is selected, which is physically

realistic but still provides some leeway for frequency deviations between the spectra.

Four different spectral similarity measures have been employed in accordance with previous studies.[S2–S6] The first is a simple match score (r_{msc}),

$$r_{\mathrm{msc}} = \frac{\left(\sum_i^k u_i v_i\right)^2}{\left(\sum_i^k u_i^2\right)\left(\sum_i^k v_i^2\right)} \ , \tag{S3}$$

where u and v are the k-dimensional vectors obtained for the two compared spectra. The r_{msc} corresponds to a Cauchy-Schwarz inequality in $\mathbb{R}^k$ dimensional Euclidian space which essentially is a simplified overlap. r_{msc} values range from $0 \leq r_{\mathrm{msc}} \leq 1$, where unity denotes a perfect match. If not stated otherwise, the r_{msc} is our default unit of measurement for spectral comparison.

The second measure used is the Euclidean norm (r_{euc}),

$$r_{\mathrm{euc}} = \left(1.0 + \frac{\sum_i^k (u_i - v_i)^2}{\sum_i^k (v_i)^2}\right)^{-1} \ . \tag{S4}$$

The third measure is the Pearson correlation coefficient (r_{pcc}), which is similar to the r_{msc},

$$r_{\mathrm{pcc}} = \frac{\sum_i^k (u_i - \bar{u})(v_i - \bar{v})}{\sqrt{\sum_i^k (u_i - \bar{u})^2}\sqrt{\sum_i^k (v_i - \bar{v})^2}} \ , \tag{S5}$$

with the mean values $\bar{u}$ and $\bar{v}$ for u and v. Both the r_{msc} and r_{pcc} are linear correlation measures that are based on the Cauchy-Schwarz inequality. And finally, the fourth measure is the Spearman rank correlation coefficient (r_{scc})

$$r_{\mathrm{scc}} = 1.0 - \frac{6\sum_i^k (rg(u_i) - rg(v_i))^2}{k\,(k^2 - 1)} \ , \tag{S6}$$

where $rg(u_i)$ and $rg(v_i)$ are the respective ranks of u_i and v_i.

In the literature, other measures have been proposed and adapted for various purposes,[S7] however, in the context of spectra comparison, Henchel et al.[S8] suggested that the different similarity scores have different advantages and disadvantages and can be used complementary, which is in line with our own previous work.[S6]

2 Dataset composition

The IR7193 dataset was originally[S6] proposed by obtaining all experimentally available gas-phase IR spectra of the NIST database.[S9] The distribution of system sizes in IR7193 is shown in Figure S1. The occurrence of different elements is visualized in Figure S2. Systems

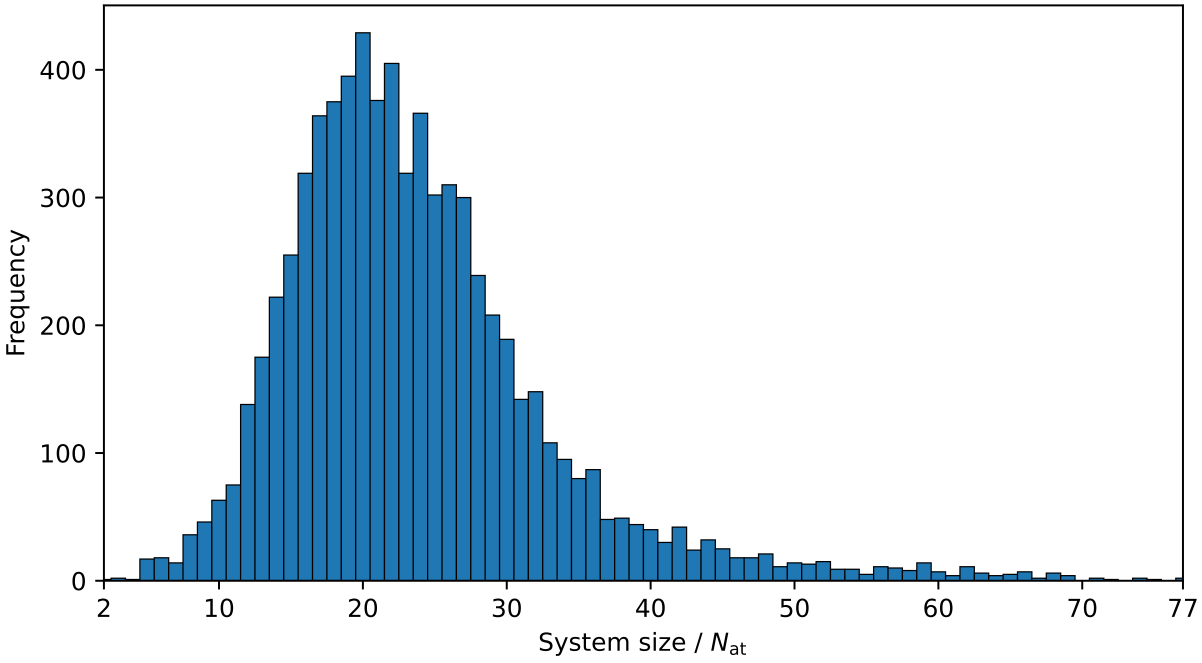

Figure S1: System sizes in IR7193.

with the elements HCNO are present in abundance. The smallest subset are phosphorous containing systems with exactly 100 molecules.

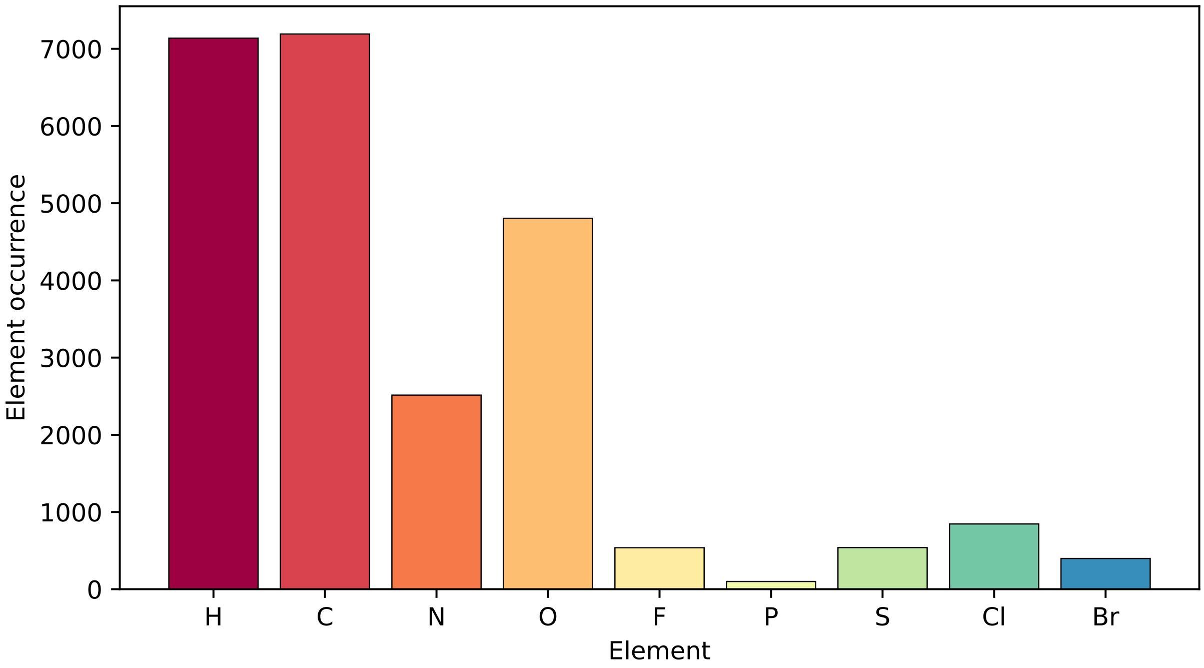

Figure S2: Element occurrence histogram for the IR7193 database.

3 Molecular geometries at ωB97M-D3(BJ) level

As an additional perspective on the molecular geometry quality, all structures of IR7913 were reoptimized at the ωB97M-D3(BJ)/def2-TZVPP level of theory. This reference level only differs from the SPICE set[S10] reference by the additional diffuse functions in the basis set, which were omitted here for sake of computational performance. However, according to best DFT practices,[S11] it is reasonable to assume that differences between the two basis sets are insignificant for optimized geometries. Possibly much greater differences can be expected between B3LYP-3c and ωB97M-D3(BJ)/def2-TZVPP. Despite employing some hybrid-level DFT in both cases, the basis sets differ in cardinal number, which is a major factor for both computational cost and performance. The range-separation of exchange in ωB97M-D3 further represents a key difference which influences the molecular geometries.

A comparison of the performance of B3LYP-3c and MACE-OFF23(large) is shown in Figure S3. The full evaluation of the MACE and xTB low-cost potentials, analogous to the main article, can be found in Tab. S1, Tab. S2 and Figure S4.

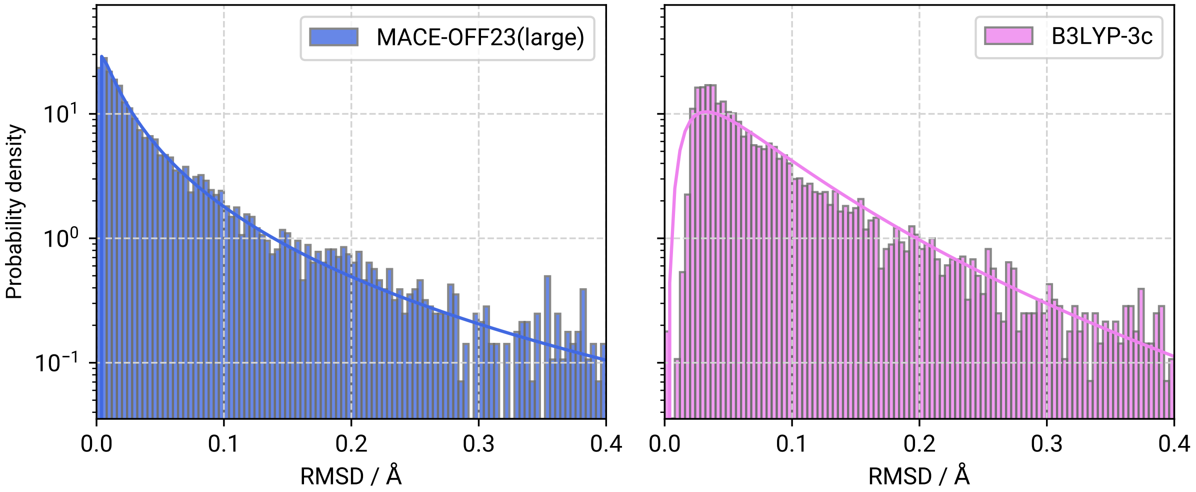

Figure S3: Histograms and fitted log-normal distributions for Cartesian RMSDs calculated between the ωB97M-D3(BJ)/def2-TZVPP reference and the MACE-OFF23(large) and B3LYP-3c minima for the IR7193 set. All plots use a logarithmic scale to emphasize the distribution tails.

Table S1: Mean, median, and standard deviation (SD) for Cartesian RMSDs calculated between the ωB97M-D3(BJ)/def2-TZVPP reference and B3LYP-3c, MACE-OFF23(small/medium/large) and GFNn-xTB/FF optimized structures of IR7193. All values are in Ångström. Narrow distributions indicate better performance.

	**B3LYP-3c**	**MACE-OFF23 model**			**GFN2-xTB**	**GFN1-xTB**	**GFN-FF**
		**small**	**medium**	**large**			
**Mean**	0.0951	0.0878	0.0704	0.0642	0.1351	0.1504	0.2419
**Median**	0.0571	0.0419	0.0308	0.0262	0.0686	0.0800	0.1369
**SD**	0.1177	0.1379	0.1182	0.1141	0.1864	0.2060	0.2668

Table S2: Percentage of structures for IR7193 falling within the specified Cartesian RMSD threshold at a given level of theory.

**RMSD [Å]**	**B3LYP-3c**	**MACE-OFF23 model**			**GFN2-xTB**	**GFN1-xTB**	**GFN-FF**
		**small**	**medium**	**large**			
≤ 0.2	90.31%	88.84%	91.77%	92.88%	80.88%	77.78%	60.02%
≥ 0.5	1.46%	2.03%	1.33%	1.24%	5.24%	6.02%	14.90%
≥ 1.0	0.26%	0.35%	0.22%	0.24%	0.71%	1.13%	2.14%

Two main effects are observed when referring to ωB97M-D3(BJ)/def2-TZVPP minima instead of B3LYP-3c in Figure S4: First, the log-normal distribution peak is notably left-shifted for all low-cost potentials, and secondly, the distribution tails are longer in each

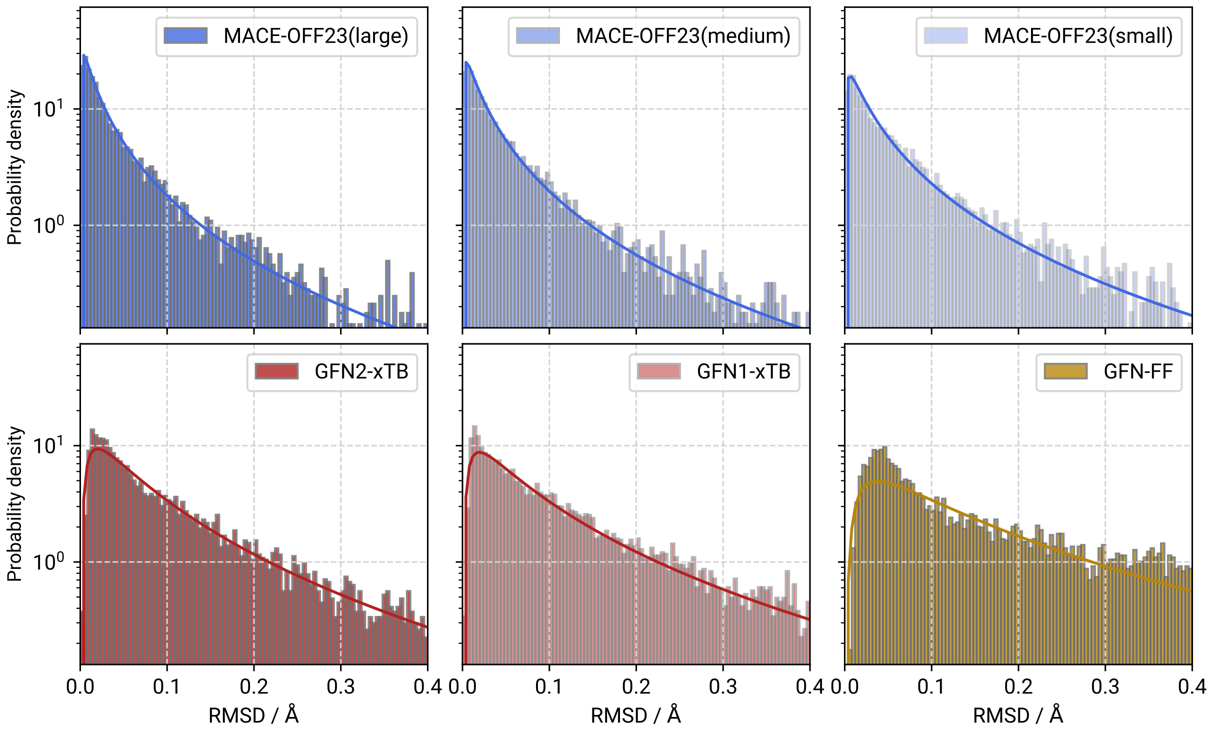

Figure S4: Histograms and fitted log-normal distributions for Cartesian RMSDs calculated between the ωB97M-D3(BJ)/def2-TZVPP minima and corresponding optimized molecular structures at B3LYP-3c, MACE-OFF23(small/large) and GFNn-xTB/FF levels of theory for the IR7193 set. All plots use a logarithmic scale to emphasize the distribution tails.

case. This is reflected by both table S1 and S2, in particular for the MACE-OFF23 models which show a similar a mean, lower median and higher standard deviation compared to the B3LYP-3c-based results from the main article. Here, while cases with RMSDs below 0.2 Å are fewer compared to the previous comparison, the percentage of even lower RMSD pairs *within* the 0.2 Å window increased leading to the left-shifted log-normal distribution maxima. Since MACE-OFF23 is trained to ωB97M-D3(BJ) data via the SPICE set, this performance is not unexpected although the overall errors with regard to either DFT reference seems small. GFNn-xTB/FF, on the other hand, show slightly larger disagreements with ωB97M-D3(BJ)/def2-TZVPP compared to the previously evaluated B3LYP-3c. A possible explanation, in particular for the xTB methods, is that these share some similarities with the "3c" method family and have been parametrized to the PBEh-3c[S12] and B97-3c[S13] data.[S14] Finally, comparing B3LYP-3c and ωB97M-D3(BJ)/def2-TZVPP, one can observe some per-

formance differences between the two DFT methods. Overall, B3LYP-3c performs *worse* than MACE-OFF23(medium/large) which becomes apparent from Figure S3, but better than GFNn-xTB/FF and MACE-OFF23(small). As noted above, we expect these differences to be a result of the much larger basis set and the range-separated exchange present in ωB97M-D3(BJ)/def2-TZVPP, making the latter a generally better choice for a reference level of theory. Unfortunately, the large difference in computational cost between the two DFT methods currently prevents us from calculating frequencies at the ωB97M-D3(BJ) level at present time, as this goes much beyond the task of obtaining molecular geometries. We are planning a revision for future work.

4 Dipole moment benchmarking

Our reference method for dipole moments in the main manuscript is ωB97M-D3(BJ)/def2-TZVPPD, since MACE is trained to this level of theory via the SPICE dataset.[S10] For a very few number of cases the smaller basis set def2-TZVPP had to be employed to circumvent SCF convergence issues. In total, this affected 42 out of 7193 molecules (0.58 %).

To estimate the performance of these reference methods to even high-level reference data, a benchmark set of CCSD(T)/CBS extrapolated dipole moments of small molecules was used. The original set was published by Head-Gordon and coworkers,[S15] however, we refer to a slightly smaller subset of 114 molecules which excludes transition metals, as used by Zapata et al.[S16] This benchmark set is abbreviated as MHG114 in the following. The respective data is shown in Figure S5 and Tables S3 and S4.

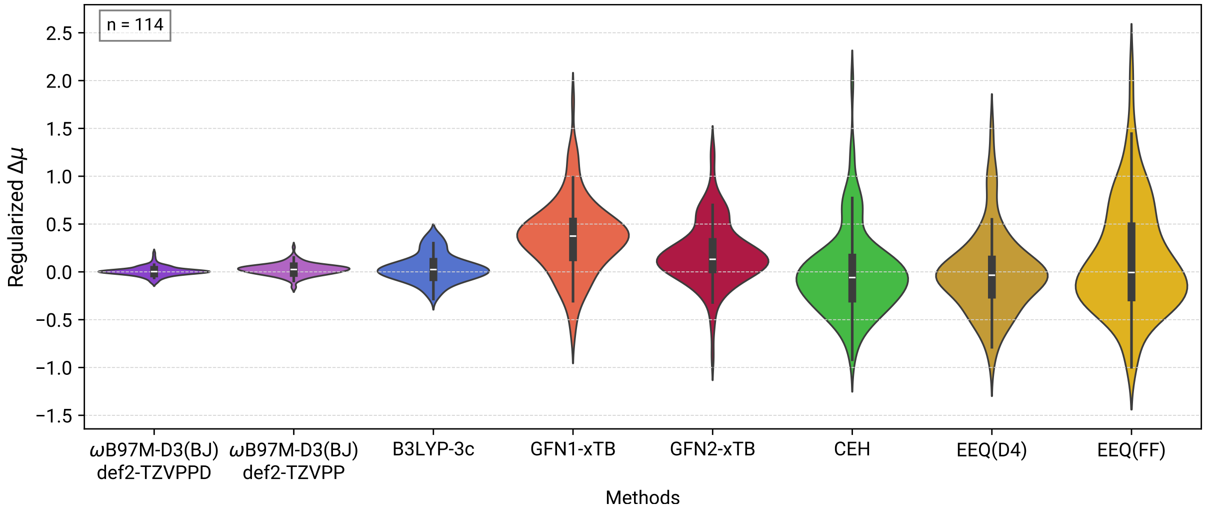

Figure S5: Violin plots for regularized dipole differences $\Delta\mu$ obtained from deviations between reference CCSD(T)/CBS dipoles of the MHG114 set[S15,S16] and dipole moments calculated at the shown levels of theory.

Overall, a clear correlation with the level of theory can be observed for the tested methods can be observed. With an $\Delta\mu$ MAD of only 3.27%, ωB97M-D3(BJ)/def2-TZVPPD shows excellent performance on the MHG114 set and surely is sufficient to serve as our reference for further comparisons. The basis set size slightly affects (worsens) this performance, shown through ωB97M-D3(BJ)/def2-TZVPP with an MAD of 4.49%. B3LYP-3c, with an MAD

Table S3: Errors in units of regularized dipole moment deviations for DFT and SQM methods tested on the MHG114 set of CCSD(T)/CBS dipole moments.

	ωB97M-D3(BJ)/def2-TZVPPD	ωB97M-D3(BJ)/def2-TZVPP	B3LYP-3c	GFN1-xTB	GFN2-xTB
MD	0.0051	0.0238	0.0383	0.3553	0.1881
MAD	0.0327	0.0449	0.1087	0.2752	0.2338
RMSE	0.0475	0.0609	0.1429	0.3789	0.3209

Table S4: Errors in units of regularized dipole moment deviations for (semi-)classical and ML methods tested on the MHG114 set of CCSD(T)/CBS dipole moments.

	CEH	EEQ(D4)	EEQ(GFN-FF)	MACE-μ medium	MACE-μ small
MD	-0.0127	-0.0157	0.1266	–	–
MAD	0.3033	0.2932	0.4470	–	–
RMSE	0.4294	0.4178	0.5676	–	–

of 10.87%, provides a balanced performance that is in line with previous observations for smaller basis sets.[S16] The semiempirical GFN1-xTB and GFN2-xTB methods, as well as the semi-classical models (Table S4) show much larger deficiencies. Partly, this can be attributed to a number of open-shell systems in the benchmark set, which are either insufficiently or entirely incorrectly described at these levels of theory. This observation was already made during the GFN2-xTB method development.[S17] The MACE-μ models were not evaluated on the MHG114 set since several open-shell molecules are contained within and there is no option for treating such systems with the MLP.

Since molecules in the MHG114 are small and far from typical "use-case" molecules (e.g. drugs), we benchmarked low-cost methods on a far larger set of structures, taking ωB97M-D3(BJ) dipole moments as a reference. The benchmark set was composed out of the IR7193 set of molecules, as well as three smaller benchmark sets focusing on different conformations of molecules: MALT222,[S18] MPCONF196[S19], and 37conf8.[S20] The summary of the evaluations are given in the main manuscript. However, violin plots for regularize dipole moment differences of each individual set can be found in Figure S6.

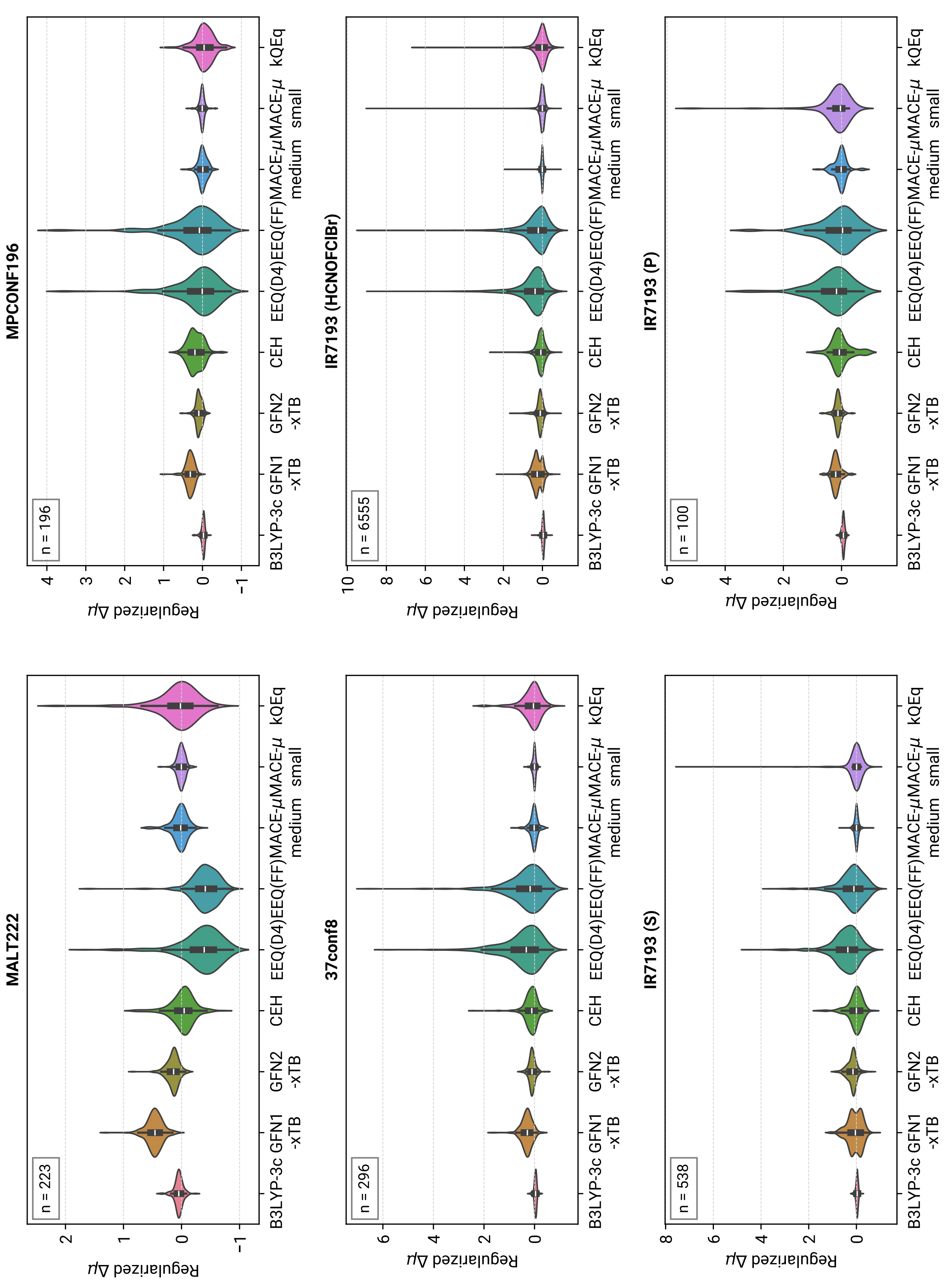

Figure S6: Violin plots for regularized dipole moment differences $\Delta\mu$, shown for a variety of benchmark subsets and methods. Reference dipole moments were obtained at the ωB97M-D3(BJ)/def2-TZVPPD level of theory. Figure rotated by 90° to fit landscape page view.

4.1 Dipole orientation

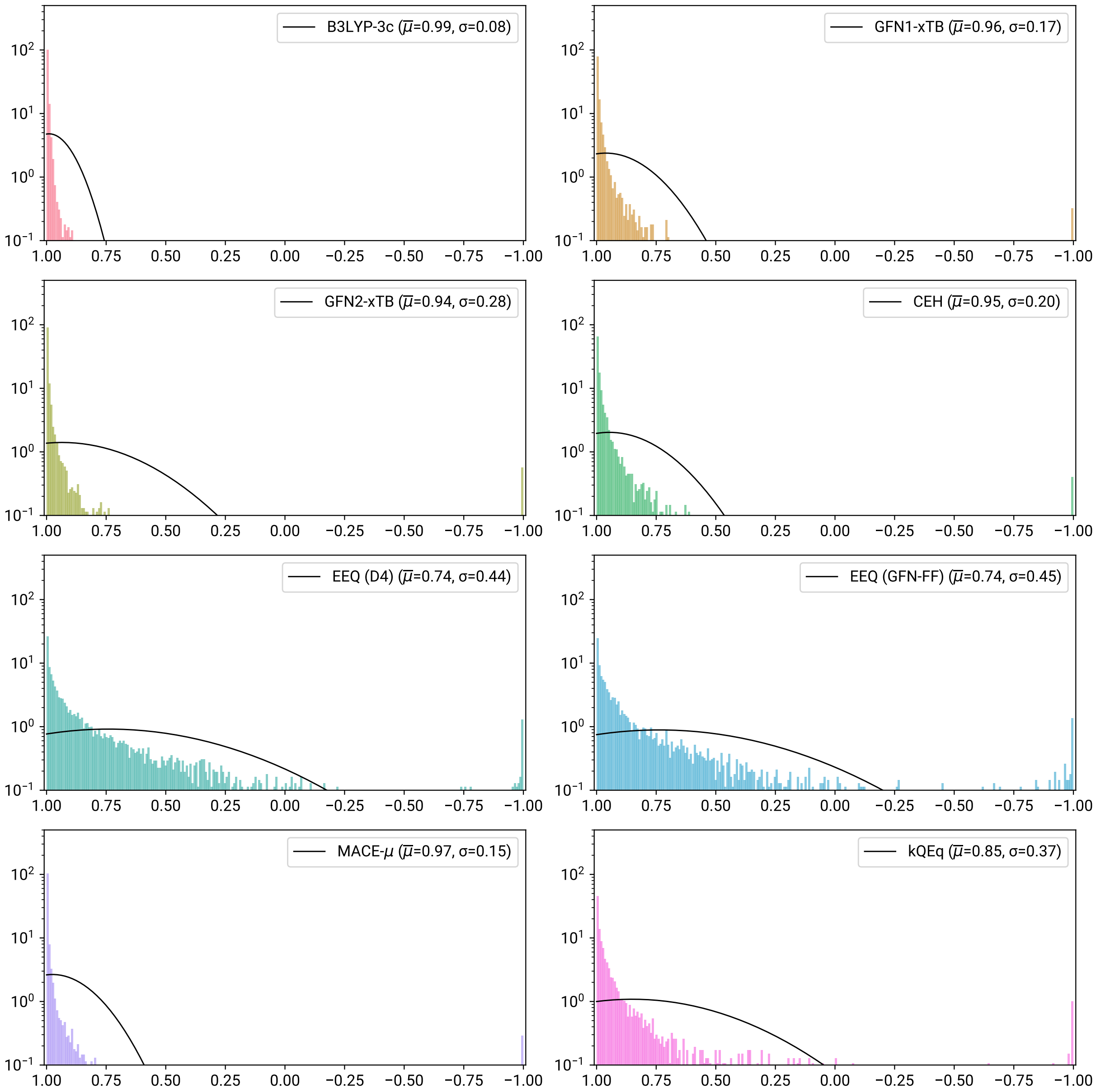

Figure S7: Histogram plots for data presented in Figure 6 of the main manuscript. Data plotted with logarithmic scales for emphasis on the distribution tails. The horizontal axes refer to the dot product between the respective method's $\hat{\mu}$ and the reference ωB97M-D3(BJ) dipole moments $\hat{\mu}^{\text{ref}}$.

4.2 Charge distribution of dodecanoic acid

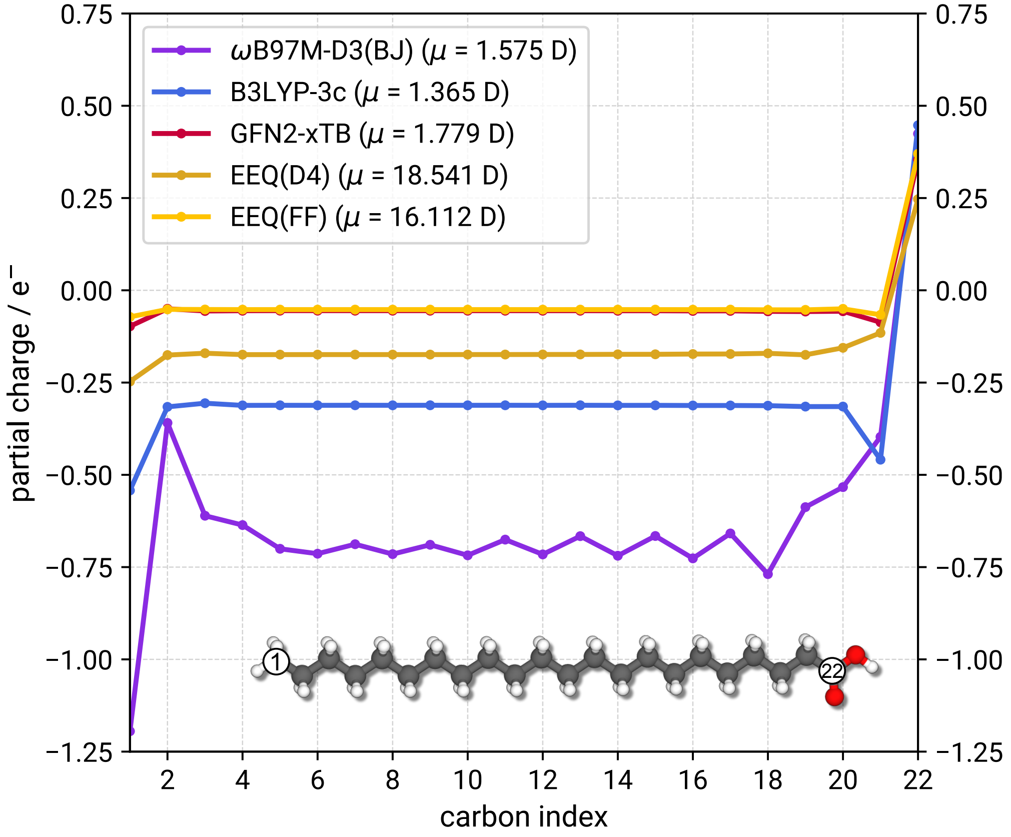

Figure S8: Distribution of partial charges on the carbon atoms of dodecaonic acid, shown for ωB97M-D3(BJ)/def2-TZVPPD, B3LYP-3c, GFN2-xTB, EEQ(D4) and EEQ(GFN-FF). Carbon atoms are numbered sequentially 1 to 22, as shown.

4.3 MACE-μ model training

The parameters used to set up the MACE-μ model closely follow those from the MACE-OFF23 paper. Fitting dipoles with the MACE architecture only requires implementing the readout with L=1 equivariance. We use 128 equivariant channels with a maximum equivariant order of L=1 and a cutoff of 6 Å, along with two interaction layers, resulting in a receptive field of 12 Å. As these parameters have been shown to provide a good mapping between atomic representations and the system's energy, they served as a strong starting point for learning the total dipole. The readout multi-layer perceptron uses 16 equivariant channels. We train directly on the total dipole moments, without using any point charge baseline, on the same dataset[S10] as the MACE-OFF series of models. A batch size of 100 provided the best trade-off between the computational cost per epoch and the rate of loss

convergence at a learning rate of 0.01. The actual training call is included below, with data paths removed:

```
python ~/source/mace/scripts/run_train.py \
    --name="SPICE_medium_dipole" \
    --train_file="(...)" \
    --valid_file="(...)" \
    --test_dir="(...)" \
    --statistics_file="(...)/statistics.json" \
    --model="AtomicDipolesMACE" \
    --num_interactions=2 \
    --num_channels=128 \
    --max_L=1 \
    --correlation=3 \
    --dipole_key='dipole' \
    --loss='dipole' \
    --weight_decay=5e-10 \
    --clip_grad=1.0 \
    --batch_size=100\
    --valid_batch_size=100 \
    --max_num_epochs=100 \
    --scheduler_patience=20 \
    --patience=50 \
    --eval_interval=1 \
    --ema \
    --error_table='DipoleRMSE' \
    --default_dtype="float64"\
    --device=cuda \
    --seed=123 \
    --restart_latest \
    --save_cpu
```

5 Timings

5.1 Timings for potential evaluations

Computational wall-times for simple potential evaluation were compared for thirteen model systems ranging in size from 84 to 1044 atoms. The model systems correspond to non-covalent clusters modelling the Miller-Urey experiment, all with a 1:1:1 ratio of H_2O, NH_3 and CH_4 molecules. All calculations were performed using a 11th Gen Intel Core i7-11800H (2.30GHz) processor and 4 shared memory threads. The documented timings are shown in Figure S9. Calculations for the MACE-OFF23(large) for systems with 888, 960, and 1044 atoms terminated early because of extensive memory requirements, exceeding the available 16 GB RAM plus 7 GB swapping partition. MACE-OFF23 timings do *not* include model initialization time. Note, even further speed-up for the MACE-OFF23 models is possible by utilizing the GPU implementation. The CPU implementation was used here to ensure direct comparability to the xTB methods. All calculations were repeated 3 times to obtain an average wall-time for each corresponding system size.

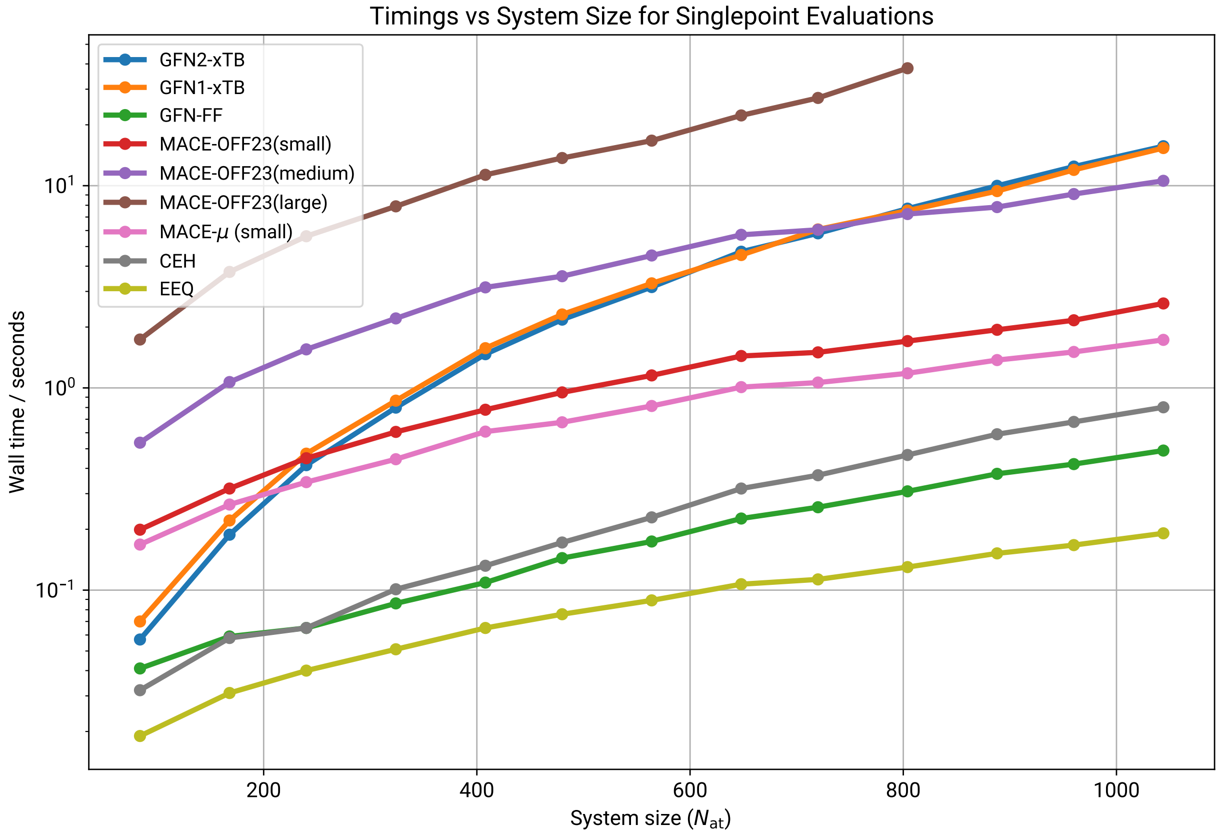

Figure S9: Singlepoint evaluation wall-times vs. system size for methods employed in this study. Note a logarithmic scale is used. The smallest system has 84 atoms, the largest system has 1044 atoms. All calculations refer to CPU timings and use identical hardware.

5.2 Timings for Hessian evaluations

Computational wall-times were investigated for a subset of IR7193, taking one molecule for each available size (2 to 77 atoms). To highlight the performance for MACE-OF23, wall-times were documented for these molecules using both the numerical Hessian implementation, and the "autograd" implementation. All calculations were performed using a 11th Gen Intel Core i7-11800H (2.30GHz) processor and a variable number of shared memory threads.

Expectantly, Hessian calculation wall-times should scale quadratically with the system size N, the molecule's number of atoms. Generalizing this relation for comparison, a simple Power-law scaling function $\mathcal{O}(aN^b)$ was fitted to the documented wall-times. The determined Power-law factors a and b for the small, medium, and large MACE models are shown in Table S5, parallelization was tested and is shown only for the MACE-OFF23(small) model in Figure S11.

Table S5: Fitted factors a and b of the Power-law scaling function $\mathcal{O}(aN^b)$ for the specified MACE-OFF23 and Hessian calculation type combinations. The data corresponds to timings given in Figure 4 in the main article. N is the system size (number of atoms), all calculations used 4 threads for shared memory (OpenMP) parallelization. Obtained on a 11th Gen Intel Core i7-11800H (2.30GHz) processor.

**MACE-OFF23 model**	**Hessian type**	a	b
small	numerical	0.0216	1.8108
medium	numerical	0.0394	2.0962
large	numerical	0.1614	2.0161
small	autograd	0.0047	2.0469
medium	autograd	0.0224	2.1469
large	autograd	0.1441	1.9613

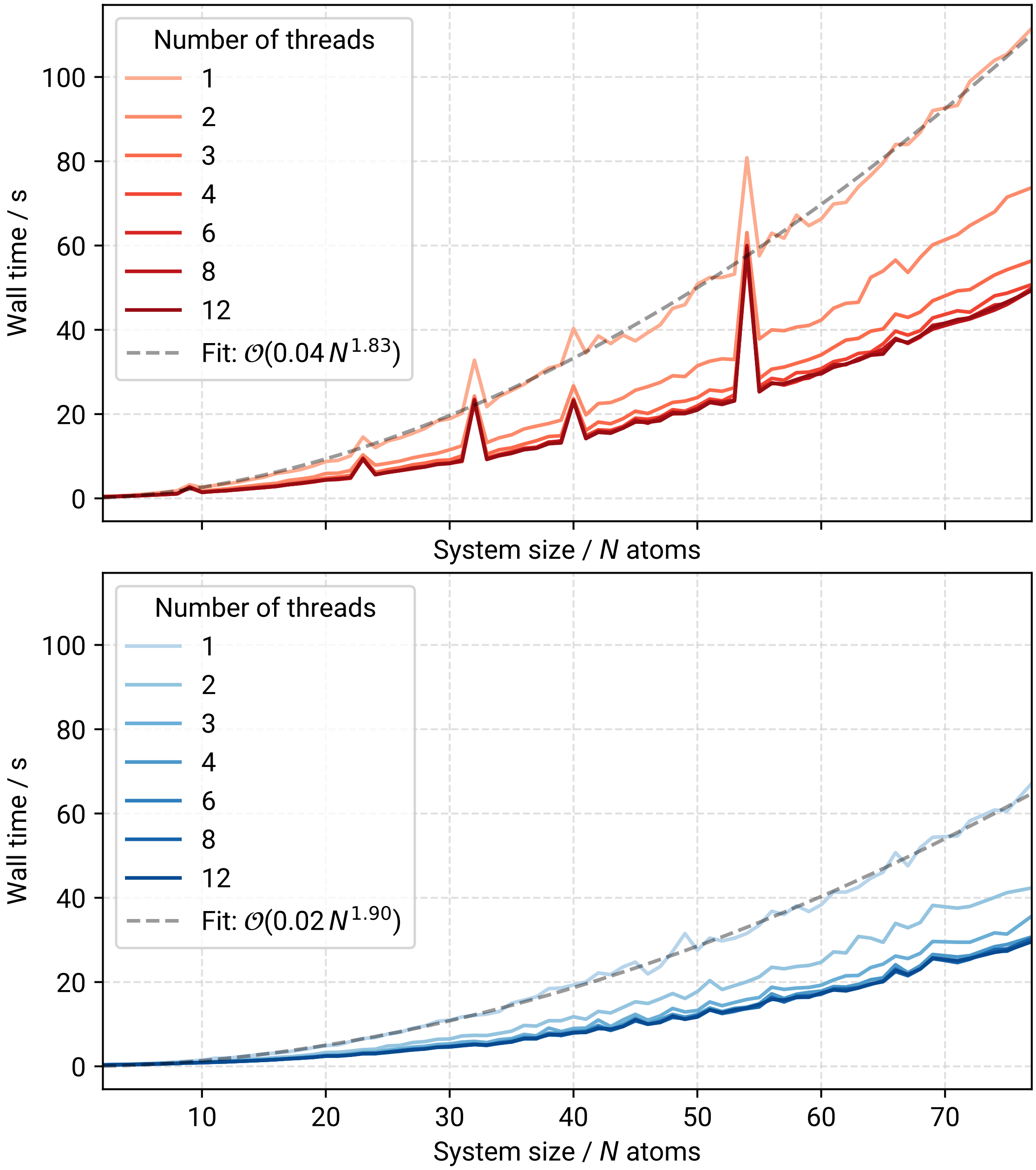

Figure S10: Shared memory parallelization (OpenMP) acceleration for numerical (top, red) and autograd (bottom, blue) Hessian calculations with the MACE-OFF23(small) model. Obtained on a 11th Gen Intel Core i7-11800H (2.30GHz) processor.

6 Problematic NIST database entries

Unfortunately, some entries in the NIST database were found to be problematic. In particular, several cases have a mismatch between the documented 3D structure that can be downloaded from NIST and the actual molecule for which the data is provided. One such example is 2-chloro-N,N-diethyl-acetamide with the ID C2315368. Here, the Cartesian coordinates offered by NIST to download show a severe chemical change including the dissociation of a H_2 molecule:

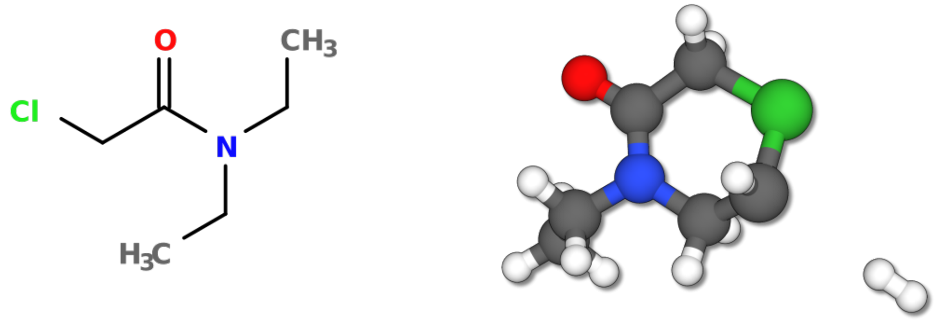

Figure S11: NIST ID C2315368, depicted as the expected Lewis structure, and the 3D coordinates directly available from the NIST database.

Another case with the same type of mismatch and dissociation within the 3D structure is Ethyl-2,5-dichlorobenzoate (C35112277). The "mirex" molecule (C2385855), which is another high error outlier for MACE-μ and was shown in Figure 7d of the main manuscript, features a broken C-C bond compared to the expected 2D structure. It is likely more such cases exist that so far went undetected. The affected NIST entries should be revised in the online database. As our main target is a theory-to-theory comparison, some of the resulting errors are mitigated because both the reference and the calculation still refer to the same structure. However, there will be a significant mismatch between the experimental and the calculated data.

Some of the affected molecules may show up as outliers in the computations, as was the case for MACE-μ, but this is not guaranteed and results otherwise are entirely agnostic to

the theory-to-theory comparison workflow. In fact, a main check was to look for the presence of imaginary modes in the DFT spectra, which all molecules passed. This makes identifying chemical misrepresentations difficult, especially in large datasets as the present one.

7 Experimental IR spectra comparison

For completeness, calculated spectra were compared to experimental gas-phase spectra taken from the NIST database.[S9] The resulting average matchscores are presented for raw and scaled frequencies in Figures S12 and S13, respectively. Note the following "pitfalls" in comparing to the experiment (applying to this study but also in general):

- Conformational effects are entirely excluded from the comparison, meaning the simulated spectrum may correspond to a different conformation. Furthermore, a few cases have chemically wrong 3D coordinates documented in the database, as discussed in section 6 above.

- Experimental spectra can have noise, which directly influences the comparison metric. Spectral smoothing procedures, e.g. Savitzky-Golay filtering, were not applied.

- There is uncertainty with regards to the measurement conditions, in particular the temperature. Accounting to some of the conformational effects, a different conformation or multiple conformations may be populated.

- The JDX files obtained from NIST have a limited resolution of either 2 cm^{-1} or 4 cm^{-1}, and can be cut off at different frequency ranges. Often, the low cm^{-1} range (below 450 cm^{-1}) is not present in the measurement. Matchscore metrics are calculated at a resolution of 1 cm^{-1}, requiring the experimental spectrum to be interpolated. Both experimental and calculated spectra must be cut to the same range.

Employing a theory-to-theory comparison effectively circumvents these issues and allows for better control of the reference data quality.

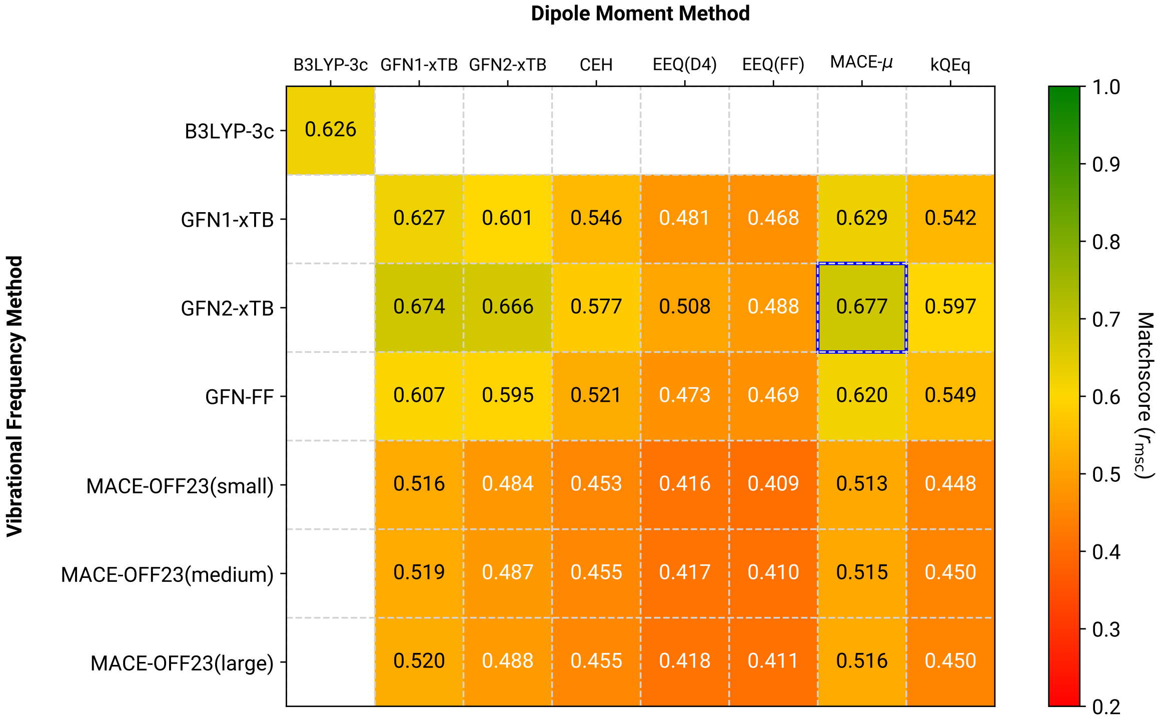

Figure S12: Average r_{msc} comparison for different frequency/dipole method combinations. Reference data refers to experimental gas-phase IR spectra taken from the NIST database. Neither of the tested methods employs any kind of scaling to the harmonic frequencies. The overall highest average matchscore is marked by a blue outline.

Results in Figure S12 for raw IR spectra predicted via the DHA demonstrate mostly the influence of wrong frequencies, that is the harmonic frequencies rather than anharmonic (experimental) ones. Expectantly, the highest observed average matchscore is 0.677 for the combination of GFN2-xTB with MACE-μ. B3LYP-3c, as our theoretical reference, only shows a mediocre matchscore of 0.626, which must mainly be attributed to the lack of frequency scaling, or rather lack of compensation for vibrational anharmonicity. As discussed in the main text, the semiempirical tight-binding methods are rather insensitive to frequency scaling, which is again reflected by this outcome.

Significant improvements can be made to the spectra matchscores by appropriately scaling the harmonic frequencies. The corresponding average r_{msc} for B3LYP-3c and the MACE-OFF23 MLPs see substantial improvement upon including this correction. In fact, the best

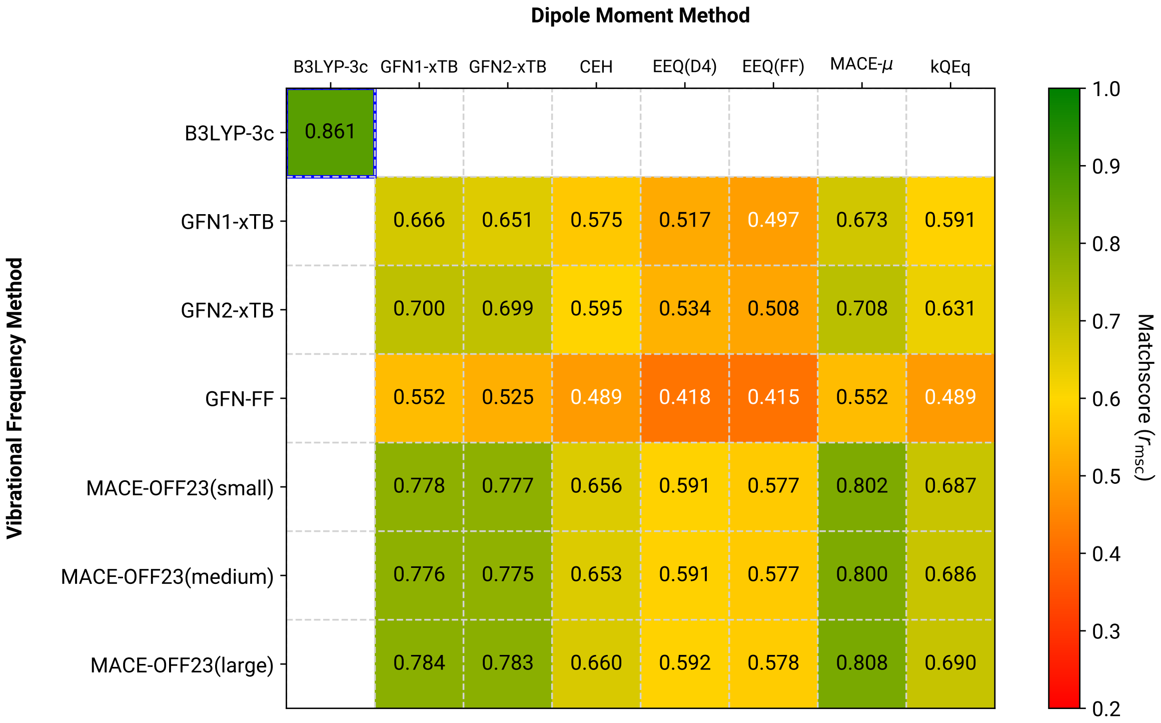

Figure S13: Average $r_{\rm msc}$ comparison for different frequency/dipole method combinations. Reference data refers to experimental gas-phase IR spectra taken from the NIST database. All tested methods employ scaling to the harmonic frequencies. The overall highest average matchscore is marked by a blue outline.

performing method, with an average matchscore of 0.861 is B3LYP-3c, with all MACE-OFF23 MLPs following close behind at $r_{\rm msc}$ of 0.800 to 0.808. The GFNn-xTB methods experience only moderate improvement due to scaling of frequencies, which is expected. The frequency scaling for the classical GFN-FF force field is asystematic, in most cases even worsening the average matchscore.

Part of the performance difference between B3LYP-3c and the next best method, MACE-OFF23(large)/MACE-μ, is intrinsic to the methods. This difference is apparent when comparing the histograms of observed matchscores for the two methods, as provided in Figure S14. Both the raw and the frequency scaled versions of the results show on average higher matchscores for B3LYP-3c than for MACE. Distributions for the scaled results are much more narrow than their raw counterpart, attesting to a systematic nature of the error

in harmonic frequencies. Since this frequency error is present in both the B3LYP and MACE results, other, methodological errors, for example stemming from the geometry optimization or the intensity prediction, must be responsible for the remaining difference.

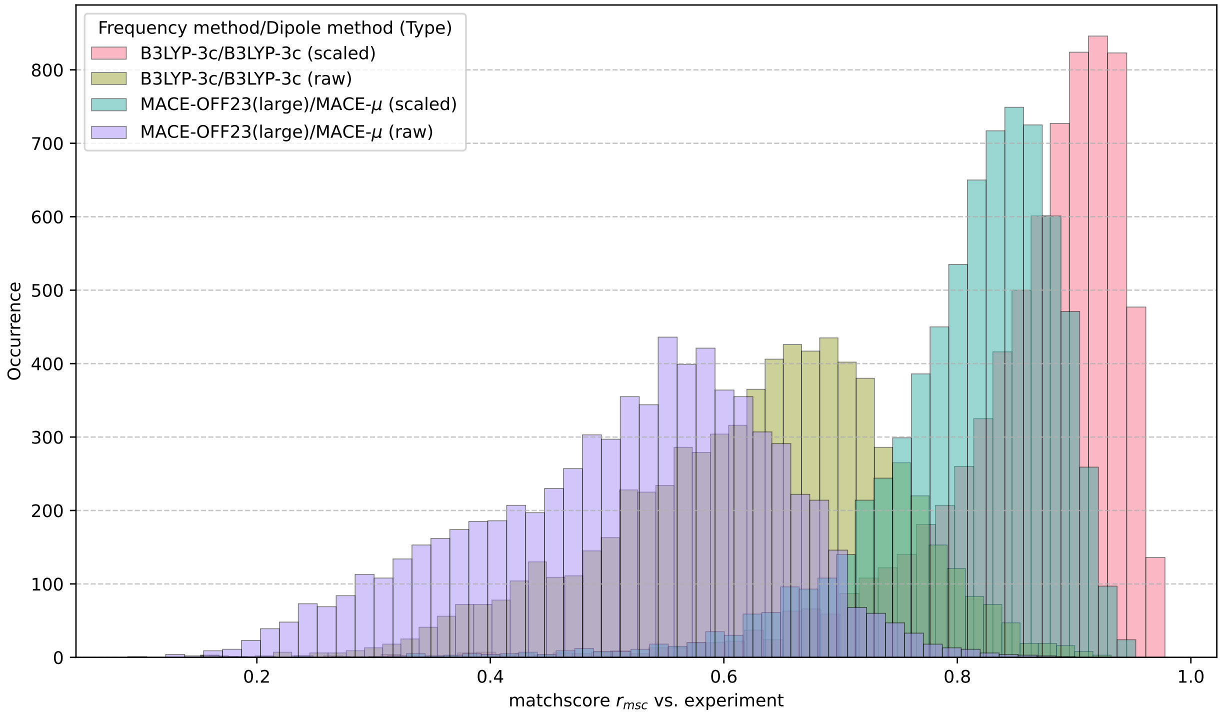

Figure S14: r_{msc} histogram for B3LYP-3c and MACE-OFF23(large)/MACE-μ. Reference data refers to experimental gas-phase IR spectra taken from the NIST database. Application of frequency scaling is denoted by the legend labels.

References

(S1) Chu, P. M.; Guenther, F. R.; Rhoderick, G. C.; Lafferty, W. J. The NIST Quantitative Infrared Database. *J. Res. Natl. Inst. Stand. Technol.* **1999**, *104*, 59–81.

(S2) Baumann, K.; Clerc, J. Computer-assisted IR spectra prediction — linked similarity searches for structures and spectra. *Anal. Chim. Acta* **1997**, *348*, 327–343.

(S3) Tan, X.; Chen, X.; Song, S. A computational study of spectral matching algorithms for identifying Raman spectra of polycyclic aromatic hydrocarbons. *J. Raman Spectrosc.* **2017**, *48*, 113–118.

(S4) Vrancic, C.; Petrich, W. Effective Fragment Potential Study of the Influence of Hydration on the Vibrational Spectrum of Glucose. *J. Phys. Chem. A* **2011**, *115*, 12373–12379.

(S5) Zapata, F.; García-Ruiz, C. The discrimination of 72 nitrate, chlorate and perchlorate salts using IR and Raman spectroscopy. *Spectrochim. Acta A* **2018**, *189*, 535–542.

(S6) Pracht, P.; Grant, D. F.; Grimme, S. Comprehensive Assessment of GFN Tight-Binding and Composite Density Functional Theory Methods for Calculating Gas-Phase Infrared Spectra. *J. Chem. Theory Comput.* **2020**, *16*, 7044–7060.

(S7) Penchev, P. N.; Sohou, A. N.; Andreev, G. N. Description and Performance Analysis of an Infrared Library Search System. *Spectroscopy Letters* **1996**, *29*, 1513–1522.

(S8) Henschel, H.; Andersson, A. T.; Jespers, W.; Mehdi Ghahremanpour, M.; van der Spoel, D. Theoretical Infrared Spectra: Quantitative Similarity Measures and Force Fields. *J. Chem. Theory Comput.* **2020**, *16*, 3307–3315.

(S9) Linstrom, E. P.; Mallard, W. NIST Chemistry WebBook, NIST Standard Reference Database Number 69. `https://webbook.nist.gov/chemistry/`, accessed December 18, 2020.

(S10) Eastman, P.; Behara, P. K.; Dotson, D. L.; Galvelis, R.; Herr, J. E.; Horton, J. T.; Mao, Y.; Chodera, J. D.; Pritchard, B. P.; Wang, Y.; De Fabritiis, G.; Markland, T. E. SPICE, A Dataset of Drug-like Molecules and Peptides for Training Machine Learning Potentials. *Sci. Data* **2023**, *10*, 11.

(S11) Bursch, M.; Mewes, J.-M.; Hansen, A.; Grimme, S. Best-Practice DFT Protocols for Basic Molecular Computational Chemistry**. *Angew. Chem. Int. Ed.* **2022**, *61*, e202205735.

(S12) Grimme, S.; Brandenburg, J. G.; Bannwarth, C.; Hansen, A. Consistent structures and interactions by density functional theory with small atomic orbital basis sets. *J. Chem. Phys.* **2015**, *143*, 054107.

(S13) Brandenburg, J. G.; Bannwarth, C.; Hansen, A.; Grimme, S. B97-3c: A revised low-cost variant of the B97-D density functional method. *J. Chem. Phys.* **2018**, *148*, 064104.

(S14) Bannwarth, C.; Caldeweyher, E.; Ehlert, S.; Hansen, A.; Pracht, P.; Seibert, J.; Spicher, S.; Grimme, S. Extended tight-binding quantum chemistry methods. *WIREs Comput. Mol. Sci.* **2021**, *11*, e01493.

(S15) Hait, D.; Head-Gordon, M. How Accurate Is Density Functional Theory at Predicting Dipole Moments? An Assessment Using a New Database of 200 Benchmark Values. *J. Chem. Theory Comput.* **2018**, *14*, 1969–1981.

(S16) Zapata, J. C.; McKemmish, L. K. Computation of Dipole Moments: A Recommendation on the Choice of the Basis Set and the Level of Theory. *J. Phys. Chem. A* **2020**, *124*, 7538–7548.

(S17) Bannwarth, C.; Ehlert, S.; Grimme, S. GFN2-xTB – An Accurate and Broadly Parametrized Self-Consistent Tight-Binding Quantum Chemical Method with Multi-

pole Electrostatics and Density-Dependent Dispersion Contributions. *J. Chem. Theory Comput.* **2019**, *15*, 1652–1671.

(S18) Marianski, M.; Supady, A.; Ingram, T.; Schneider, M.; Baldauf, C. Assessing the Accuracy of Across-the-Scale Methods for Predicting Carbohydrate Conformational Energies for the Examples of Glucose and α-Maltose. *J. Chem. Theory Comput.* **2016**, *12*, 6157–6168.

(S19) Řezáč, J.; Bím, D.; Gutten, O.; Rulíšek, L. Toward Accurate Conformational Energies of Smaller Peptides and Medium-Sized Macrocycles: MPCONF196 Benchmark Energy Data Set. *J. Chem. Theory Comput.* **2018**, *14*, 1254–1266.

(S20) Sharapa, D. I.; Genaev, A.; Cavallo, L.; Minenkov, Y. A Robust and Cost-Efficient Scheme for Accurate Conformational Energies of Organic Molecules. *ChemPhysChem* **2019**, *20*, 92–102.
